# Discovering Early‐Stage Gas Generation Kinetics Enables Thermal Runaway Early Warning in Lithium‐Ion Batteries

**DOI:** 10.1002/advs.76433

**Published:** 2026-07-09

**Authors:** Jiabo Zhang, Qianzhen Guo, Shuaiqi Liu, Shuai Li, Xianghao Kong, Dong Han, Zhen Huang

**Affiliations:** ^1^ Key Laboratory for Power Machinery and Engineering of the Ministry of Education Shanghai Jiao Tong University Shanghai China; ^2^ Shanghai Non‐carbon Energy Conversion and Utilization Institute Shanghai China

**Keywords:** artificial intelligence, battery safety, early warning, gas generation kinetics, lithium‐ion battery, operando gas diagnostics, thermal runaway

## Abstract

Early warning of thermal runaway (TR) in lithium‐ion batteries remains constrained, as conventional indicators emerge after irreversible failure. Here, we establish a novel framework to advance gas‐based early warning by uncovering the earliest chemically driven processes preceding heat accumulation (HA). Operando gas measurements reveal a previously unrecognized chemical activation (CA) stage prior to HA, where coupled electrolyte–electrode reactions initiate at 60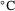, causing continuous trace gas accumulation without measurable temperature rise. To elucidate the underlying mechanisms, a physics‐informed gas generation kinetics network (GGKNet) is developed by coupling chemical reaction kinetics with gas adsorption‐desorption, electrolyte evaporation, and lithium–involved reactions. The framework quantitatively captures species‐resolved gas generation across states of charge and reconstructs pathway‐resolved kinetics during CA and HA stages. Validations under TR scenarios demonstrate that GGKNet achieves superior prediction of gas onset timing, temporal evolution, and spatial distribution, thereby translating kinetic‐level insights into guidance for practical early warning systems.

## Graphical abstract

Operando gas diagnostics reveal a previously unrecognized chemical activation (CA) stage during thermal runaway in lithium‐ion batteries. A physics‐informed gas generation kinetics network (GGKNet) is developed to reconstruct reaction pathways and physical models automatically. Validation shows that GGKNet predicts gas evolution and spatial distribution accurately, providing quantitative guidance for practical early warning.

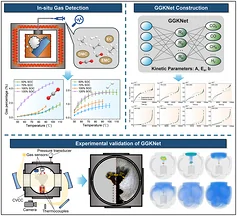

## 1 Introduction

Lithium‐ion batteries (LIBs) underpin the rapid electrification of transportation and the widespread adoption of portable and stationary battery energy storage systems (BESSs), owing to their high energy density, low self‐discharge rate, and excellent cycling performance [1]. As LIBs are increasingly deployed at larger scales and under more demanding operating conditions [2, 3], safety has emerged as a critical constraint on their sustainable development. This challenge is primarily attributed to the highly reactive nature of their internal components, including oxygen‐releasing cathode materials, lithiated graphite anodes, and flammable organic electrolytes. Under various abuse conditions, these active materials can trigger cascades of coupled chemical reactions [4, 5]. Such reactions result in the substantial generation of heat and flammable gases, which in turn lead to fire or explosion incidents, a phenomenon referred to as thermal runaway (TR) [6, 7].

To mitigate the hazards associated with TR in LIBs, effective early warning is essential to enable timely intervention before catastrophic failure occurs. TR is a progressive process involving a sequence of physicochemical transformations, typically evolving through a mild heat accumulation (HA) stage and ultimately transitioning into a violent runaway stage [8, 9]. As the violent runaway stage unfolds on an extremely short timescale [9], effective early warning must rely on signals that emerge during, or prior to, the HA stage. To this end, a variety of signals for early warning have been explored, such as temperature [10], electrical [11], mechanical [12], and gas signals [13, 14]. Among these, gas‐based warning signals have attracted increasing attention due to their high sensitivity to early‐stage chemical reactions, enabling earlier identification of instability at both the cell and system levels [15, 16]. Existing gas‐based warning approaches can be broadly categorized into external sensing methods [17, 18], which detect gases after cell venting, and internal sensing strategies that directly probe gas evolution inside the cell [19, 20]. While external sensing is inherently constrained by the opening of the safety valve, recent studies have demonstrated that internal gas sensing can detect the generation of gases such as H_2_ [21], CO_2_ [22], CH_4_ [23], prior to the onset of the HA stage, substantially earlier than detectable temperature rise. In addition, in situ gas chromatography (GC) measurements [24], thermogravimetric‐mass spectrometry (TG‐MS) [25], and in situ Raman detection [26] also show that micro‐abuse conditions, such as slight overcharge or overheating, can induce gas generation and accumulation inside the cell before the HA stage. However, this stage has not been clearly defined, with its gas‐generation kinetics, relationship to and differences from HA‐stage gas generation, and influence on the overall TR process remaining insufficiently understood. Since the period before the HA stage provides the best opportunity for safety warning and active intervention, reliable gas‐based warning strategies during this period require a comprehensive understanding of gas generation mechanisms, including their dependence on battery chemistry and state of charge (SOC) [27, 28].

Growing experimental evidence from operando and post‐mortem measurements reveals that gas generation during TR originates from coupled electrolyte and electrode reactions [24, 29]. Nevertheless, gas evolution proceeds through a highly complex reaction network involving hundreds of potential chemical species and elementary reactions spanning multiple stages of TR [30, 31], rendering pathway identification based solely on experiments and conventional modeling challenging. Within this context, artificial intelligence (AI) [32, 33] offers a powerful means to autonomously explore unknown reaction pathways from experimental observations and identify the dominant routes governing gas evolution for early‐TR warning. From a kinetic perspective, gas formation along individual pathways can be described by Arrhenius‐type rate expressions [34, 35], and a chemical reaction network (CRN) has been recently developed to predict the total gas yield [36]. However, extending such a framework toward pathway‐resolved gas prediction remains fundamentally difficult, as the number of kinetic parameters associated with individual gas‐generation routes can reach scale $O(10^{3})$, far exceeding what can be reliably identified from limited experimental datasets [36]. Furthermore, gas generation during TR is not governed by isolated chemical reactions alone, but instead arises from a tightly coupled multiphysics process that includes gas adsorption and desorption, electrolyte evaporation, and reactions involving lithium participation [21, 37]. Consequently, present AI frameworks lack the capacity to consistently capture multi‐gas pathway evolution and to quantitatively predict gas yields across varying cell states, highlighting the necessity for modeling frameworks that explicitly incorporate physicochemical constraints and cross‐process coupling.

To overcome these challenges, we propose a novel framework to advance the understanding of early‐stage gas generation in LIB TR, combining an operando experimental platform, physics‐informed data‐driven modeling, and early‐warning validation, as shown in Figure 1. Distinct from conventional approaches that rely on offline gas measurements after severe TR events and unexplainable data‐driven models (Figure 1A), the proposed framework (Figure 1B) enables operando tracking of individual gas species and their generation mechanisms during TR. Using this framework, our new insight into the early TR pathway of LIBs reveals that a previously unrecognized “chemical activation” (CA) stage exists prior to the HA stage. During CA stage, early‐stage chemical reactions are initiated at temperatures as low as 60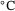 for cells with varied SOCs, leading to the accumulation of trace gaseous species (such as CO_2_, H_2_, etc.) in the absence of any measurable temperature rise. Building on these observations, a novel gas generation kinetics network (GGKNet) is developed by explicitly coupling chemical reaction kinetics with gas adsorption‐desorption, electrolyte evaporation, and lithium–involved processes. GGKNet autonomously explores previously unresolved gas‐generation pathways and reconstructs a pathway‐resolved gas evolution mechanism of H_2_, CH_4_, CO, CO_2_, C_2_H_4_, C_2_H_6_, and electrolyte vapor, over CA and HA stages, enabling quantitative prediction of early‐stage gas accumulation across different SOCs. Furthermore, the derived mechanism is integrated into a three‐dimensional computational fluid dynamics (CFD) model to predict gas accumulation and transient venting behavior, and its effectiveness for early TR warning is validated through gas‐sensing experiments.

**FIGURE 1:**
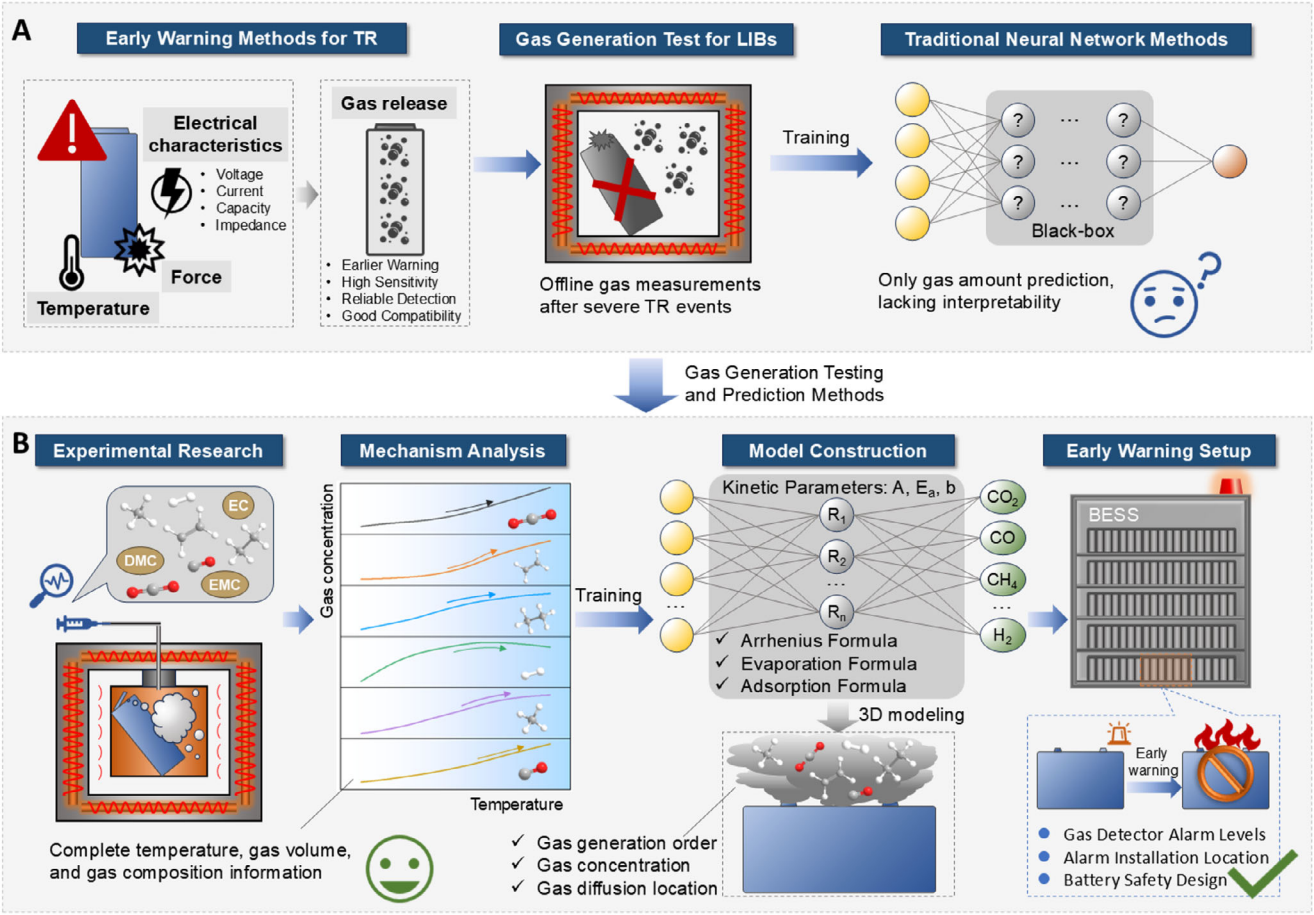
Framework for operando gas diagnostics and physics‐informed early warning of thermal runaway in lithium‐ion batteries. (A) Limitations of conventional thermal runaway studies that rely on offline gas measurements and empirical models, which fail to capture early‐stage gas generation mechanisms. (B) Schematic of the proposed framework integrating operando gas diagnostics, gas generation kinetics modeling, and engineering‐scale validation, enabling species‐resolved tracking of gas evolution for early thermal runaway warning.

## 2 Results

### 2.1 Gas Evolution During the CA Stage

To investigate gas generation prior to TR, combined Accelerating Rate Calorimetry (ARC) and gas chromatography (GC) measurements are conducted on LIBs with their top caps opened. Based on ARC characterization, gas generation is examined in the temperature range below the onset temperature of self‐heating, *T*_onset_, where no detectable HA occurs. Gas sampling and compositional analysis are conducted at successive temperature plateaus for cells with varied SOCs. Figure 2 illustrates the gas generation mechanism and the experimental results of gas generation behavior during the CA phase. As shown in Figure 2A, multiple gaseous species, including CO_2_, H_2_, CH_4_, and CO, as well as electrolyte vapor, are generated within the cells well before *T*_onset_. The continuous appearance of these gases in the absence of measurable self‐heating indicates a distinct pre‐*T*_onset_ regime, which is defined here as the CA stage. The identified chemical reactions during the CA stage are summarized in Table 1.

**TABLE 1:** Identified gas‐generation reaction pathways in LIBs during the CA stage.

Gas type	No.	Reaction equation	References
HF	1	LiPF_6_ + H_2_O ⟶ LiF + POF_3_ + 2HF	[38, 39, 46, 47, 48]
H_2_	2	HF + Li^+^ + ⟶ LiF + 0.5H_2_	[38, 39]
	3	LiH ⟶ Li + 0.5H_2_	[41, 49]
COCOccCO_2_	4	ROCO_2_Li ⟶ ROLi + CO_2_	[49, 50]
	5	ROCO_2_Li + HF ⟶ LiF + ROH + CO_2_	[46, 49, 51, 52]
	6	2ROCO_2_Li + H_2_O ⟶ Li_2_CO_3_ + 2ROH + CO_2_	[51, 52]
	7	Li_2_CO_3_ + 2HF ⟶ 2LiF + H_2_O + CO_2_	[46, 52]
CH_4_	8	C_3_H_6_O_3_(DMC) + 2Li^+^ + 2e^−^+ H_2_ ⟶ Li_2_CO_3_ + 2CH_4_	[45, 53, 54, 55, 56]
	9	C_3_H_6_O_3_(DMC) + Li^+^ + e^−^ + 0.5H_2_ ⟶ CH_3_OCO_2_Li + CH_4_	[54, 55]
CO	10	2 CO_2_+ 2Li^+^ + 2e^−^ ⟶ Li_2_CO_3_ + CO	[45, 51, 55, 56]

**FIGURE 2:**
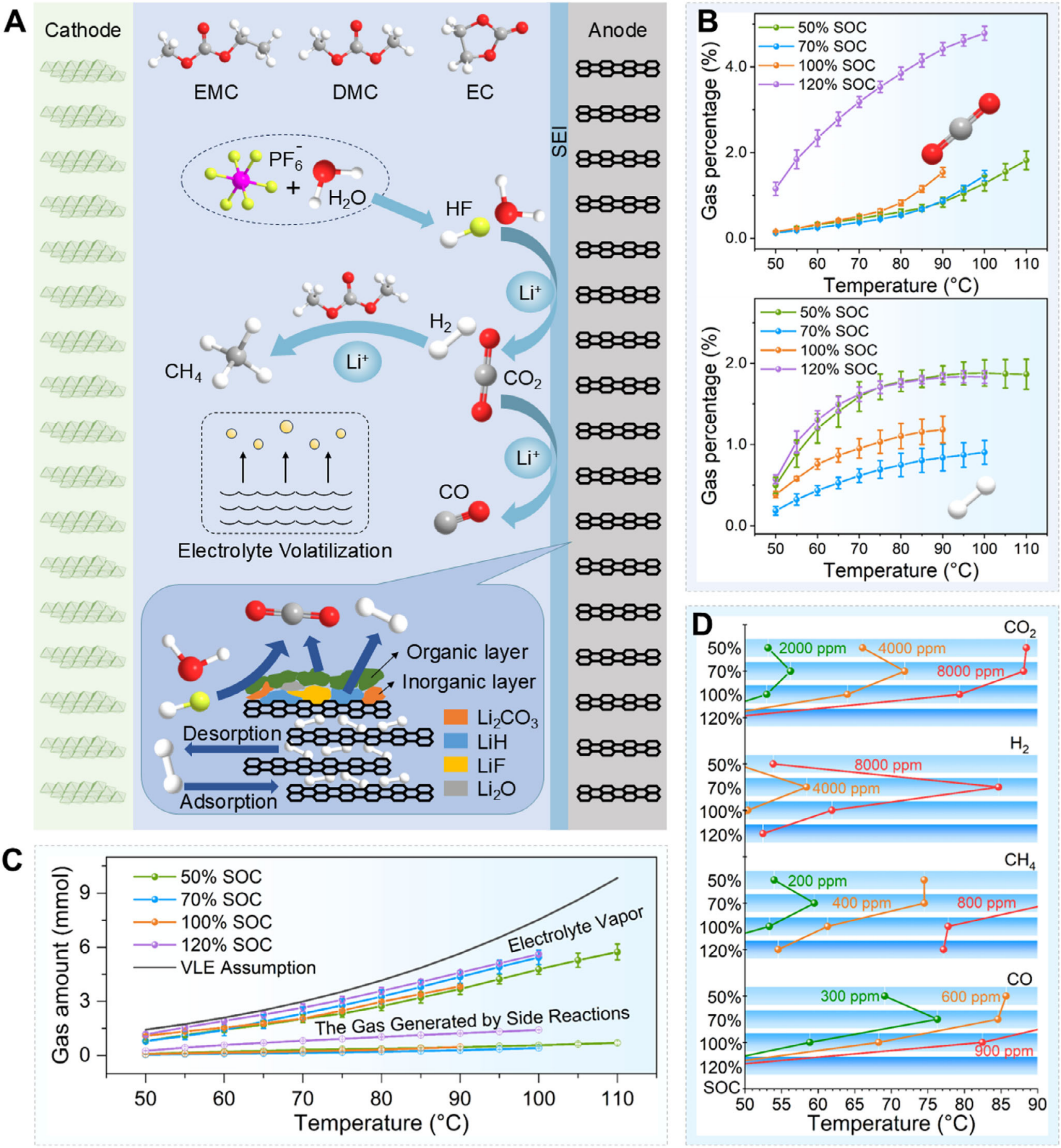
The gas generation behavior in LIBs during the CA stage. Data points represent the mean values of three independent replicates, sample size n=3; error bars indicate the experimental error. (A) The gas generation pathways in LIBs during the CA stage, highlighting the continuous volatilization of the electrolyte and concurrent side chemical reactions. (B) Evolution of major gas components during the CA stage for LIBs at 50%/70%/100%/120% SOC. Gas samples were collected from the sealed mental container, with a starting temperature of 50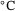 and an increasing temperature step of 5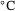. (C) Temperature‐dependent evolution of electrolyte vapor and gaseous products originating from side reactions during the CA stage, with the solid black line representing the electrolyte vapor predicted solely under the VLE assumption for 1 M LiPF_6_ in DEC/EMC/EC (1:1:1 by volume). (D) SOC‐dependent dynamic threshold design for internal gas‐based early warning during the CA stage.

Overall, the gas generation in batteries during the CA stage originates from multiple chemical processes. Specifically, the initial reaction involves the hydrolysis of LiPF_6_ to generate HF (Reaction 1), after which both trace water introduced during the battery manufacturing and assembly process [38, 39] and HF act as active reactants in subsequent gas‐generating processes. It should be noted that the generated HF is not directly quantified by GC in this study, as its exceptionally reactive nature leads to rapid consumption by other active materials upon formation. Moreover, H_2_ originates from two main pathways. One pathway is the reduction of H^+^ on the graphite anode under the influence of HF (Reaction 2). The other pathway is the decomposition of LiH within the SEI film [40] (Reaction 3). LiH is formed through the reaction between Li and H_2_ at room temperature [41], where the initial H_2_ mainly comes from the reduction of protic electrolyte oxidation species (R–H^+^) [21, 42]. The generation of CO_2_ is primarily associated with reactions between trace water or HF and organic SEI components as well as Li_2_CO_3_ (Reactions 5–7). In particular, lithium ethylene dicarbonate (LEDC) in the organic SEI layer decomposes and releases CO_2_ at approximately 50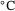 [43]. This process is further accelerated in the presence of LiPF_6_ and water [44]. Additionally, the self‐decomposition of organic SEI components also contributes to CO_2_ production (Reaction 4). In contrast, CH_4_ generation mainly originates from the decomposition of dimethyl carbonate (DMC) in the electrolyte (Reactions 8–9), during which H_2_ is consumed as a reactant. This consumption partly explains the reduced H_2_ generation rate at elevated temperatures. Finally, CO is produced through the reduction of CO_2_ on the graphite anode, accompanied by the formation of Li_2_CO_3_, which further contributes to SEI evolution (Reaction 10) [45].

Figure 2B illustrates the temperature‐dependent evolution of gas components during the CA stage for batteries with different SOCs. For clarity, only the evolution of CO_2_ and H_2_ is shown in this figure, while the concentration profiles of the remaining gaseous species are provided in Figure S5. Taking cells with 100% SOC as an example, measurable gas generation is observed as early as 50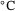, with concentrations of 0.15% CO_2_, 0.38% H_2_, and 0.013% CH_4_, whereas CO is not detected until 70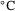. To gain deeper insights into the underlying gas generation mechanism, the temperature‐dependent gas generation rates for CA stage are further provided in Figure S12. The results reveal that for gas evolution under non‐overcharged conditions, the generation rates of CO_2_, CH_4_, and CO generally accelerate with rising temperature. In contrast, the generation rate of H_2_ displays a continuous downward trend as the temperature increases, eventually reaching a stable plateau toward the end of the CA stage. This unique early‐stage nonlinear behavior is attributed to the physical adsorption–desorption effect of H_2_ on the porous graphite anode [21]. Because the total amount of H_2_ physically trapped within the porous graphite is inherently finite, the depletion of this pre‐accumulated gas reservoir leads to a progressive decay in the desorption rate. Additionally, this behavior is further influenced by its consumption in Reactions 8 and 9, as well as the limited LiH content in the SEI film of pristine cells [41].

Gas generation during the CA stage exhibits a pronounced dependence on SOC. In general, higher SOC leads to increased production of CO_2_, CO, and CH_4_, with the highest concentrations observed at 120% SOC, followed by 100% SOC, while the profiles at 50% and 70% SOC remain comparable and at lower levels. In contrast, H_2_ generation shows a non‐monotonic dependence on SOC, with the 50% SOC cell producing H_2_ at levels comparable to those at 120% SOC. This behavior is consistent with the positive correlation between H_2_ generation and HF concentration [38], as cells at intermediate SOC are reported to produce higher HF concentrations [57, 58]. For overcharged cells at 120% SOC, the markedly elevated CO_2_ and CO levels are further associated with lithium dendrite growth and localized micro‐short circuits. As CO is generated through the reduction of CO_2_, its concentration increases correspondingly.

Figure 2C presents the decoupling of electrolyte vapor from gaseous products generated by side chemical reactions, with the electrolyte vapor amount predicted solely under the vapor–liquid equilibrium (VLE) assumption included for comparison. The results indicate that electrolyte vapor constitutes a major fraction of the total released gas during the CA stage. Under the VLE assumption, the predicted electrolyte vapor agrees well with the experimental measurements at temperatures below approximately 70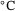, reflecting near‐equilibrium evaporation behavior enabled by the sufficiently long equilibration time. However, as the temperature increases further, the experimentally measured electrolyte vapor amount becomes consistently lower than that predicted by the VLE assumption. These observations provide evidence that electrolyte vapor may participate in early‐stage chemical processes (such as Reactions 8 and 9), thereby invalidating the conventional assumption of passive evaporation. Consequently, the results highlight the necessity of jointly accounting for electrolyte evaporation and its dynamic consumption when reconstructing early gas‐generation pathways.

During the CA stage, the internally generated gas volume remains insufficient to rupture the safety valve, corresponding to an early warning stage that cannot be detected by external gas sensors. Nevertheless, the experimental results obtained in this stage provide a quantitative basis for setting warning thresholds for internal gas sensors. As shown in Figure 2D, the dynamic thresholds for internal gas warning in batteries with different SOCs corresponding to temperature are presented. It should be noted that during normal operation, certain gas generation occurs within the battery, with CO_2_ and H_2_ reaching concentrations up to approximately 2000 ppm [42, 59], which necessitates relatively higher baseline thresholds to prevent false alarms. The results indicate that dynamic adjustment of warning thresholds based on different battery SOCs can achieve reliable earlier warnings. For instance, with a fixed H_2_ alarm threshold set at 8000 ppm, cells at 50% SOC and 100% SOC will trigger alarms at approximately 54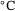 and 62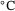, respectively, whereas cells at 70% SOC trigger an alarm only at a much higher temperature of 85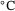. By lowering the H_2_ alarm threshold for the 70% SOC condition, the warning can be activated at a substantially earlier temperature, highlighting the advantage of dynamic, SOC‐dependent threshold design.

### 2.2 Gas Evolution During the HA Stage

As shown in Figure 3, after the CA stage, once the cell reaches *T*_onset_, more pronounced exothermic chain reactions are triggered among the active materials. These chain chemical reactions include the SEI film decomposition reaction, SEI film regeneration reaction, cathode material decomposition reaction, electrolyte solvent oxidation reaction, electrolyte decomposition reaction, and binder reaction [60, 61]. The complete gas generation mechanisms are illustrated in Figure 3A, and the relevant chemical reactions are summarized in Table 2. At approximately 90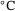 [47, 62], the metastable organic components within the SEI film begin to decompose, generating C_2_H_4_ and CO_2_ (Reaction 11). When the temperature exceeds 100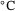 [63, 64], continued SEI degradation allows direct contact between the electrolyte and the graphite anode, leading to SEI regeneration reactions accompanied by the release of C_2_H_4_ and C_2_H_6_ (Reactions 12–15). Meanwhile, LiPF_6_ undergoes self‐decomposition at temperatures exceeding 100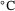 [48] (Reaction 16), and the resultant PF_5_ subsequently participates in secondary reactions at temperatures above 200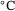 [55, 63], generating additional CO_2_ and C_2_H_4_ (Reactions 17–20). With further temperature increase beyond approximately 175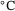, oxygen release from cathode materials becomes significant [55, 65] (Reaction 22), driving oxidative decomposition of electrolyte solvents to form CO_2_ or CO (Reactions 23–26). When the temperature exceeds 250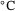 [49, 56, 66], reactions between PVDF binder and metallic lithium contribute to H_2_ generation (Reaction 27). Additionally, at temperatures surpassing 200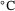 [45, 50, 55, 56, 63], other gas generation reactions are present, such as the reaction between metallic lithium and ethylene carbonate (EC), which generates CO_2_ (Reaction 21). Notably, the activation of these mechanisms during the HA stage does not suppress the gas‐generation pathways identified during the CA stage shown in Figure 2A; instead, both processes coexist and overlap as thermal runaway progresses.

**TABLE 2:** LIB gas generation reactions in the HA stage and the following violent runaway stage.

No.	Reaction equation	References
11	(CH_2_OCO_2_Li)_2_ ⟶ Li_2_CO_3_ + C_2_H_4_ + CO_2_ +0.5O_2_	[47, 55, 62, 66]
12	2Li + (CH_2_OCO_2_Li)_2_ ⟶ 2Li_2_CO_3_ + C_2_H_4_	[49, 55, 66]
13	2Li + 2C_3_H_4_O_3_(EC) ⟶ (CH_2_OCO_2_Li)_2_ + C_2_H_4_	[55, 67]
14	2Li + C_3_H_4_O_3_(EC) ⟶ Li_2_CO_3_ + C_2_H_4_	[55, 62, 66]
15	2Li + C_3_H_6_O_3_(DMC) ⟶ Li_2_CO_3_ + C_2_H_6_	[55, 62, 66]
16	LiPF_6_ ⟶ LiF + PF_5_	[48, 50, 55, 63]
17	C_2_H_5_OCOOC_2_H_5_ + PF_5_ ⟶ C_2_H_5_OCOOPF_4_ + HF + C_2_H_4_	[47, 55, 63, 64]
18	C_2_H_5_OCOOPF_4_ ⟶ POF_3_ + C_2_H_4_ + HF + CO_2_	[47, 55, 63, 64]
19	C_2_H_5_OCOOPF_4_ ⟶ POF_3_ + C_2_H_5_F + CO_2_	[47, 55, 63, 64]
20	C_2_H_5_OCOOPF_4_ + HF ⟶ PF_4_OH + C_2_H_5_F + CO_2_	[47, 55, 63, 64]
21	2Li + 2C_3_H_4_O_3_(EC) ⟶ Li‐O‐(CH_2_)_4_‐O‐Li + 2CO_2_	[45, 50, 55, 56, 63]
22	Li_0.35_(NiCoMn)_1/3_O_2_ ⟶ Li_0.35_(NiCoMn)_1/3_O_2‐y_+y/2O_2_	[5, 66, 68]
23	C_3_H_4_O_3_(EC) + 2.5O_2_ ⟶ 3CO_2_ + 2H_2_O	[5, 64, 66]
24	C_3_H_6_O_3_(DMC) + 3O_2_ ⟶ 3CO_2_ + 3H_2_O	[5, 64, 66]
25	C_3_H_4_O_3_(EC) + O_2_ ⟶ 3CO + 2H_2_O	[5, 66]
26	C_3_H_6_O_3_(DMC) + 1.5O_2_ ⟶ 3CO + 3H_2_O	[5, 66]
27	‐CH_2_‐CF_2_‐ + Li ⟶ LiF + ‐CH=CF‐ + 0.5H_2_	[51, 62, 66]

**FIGURE 3:**
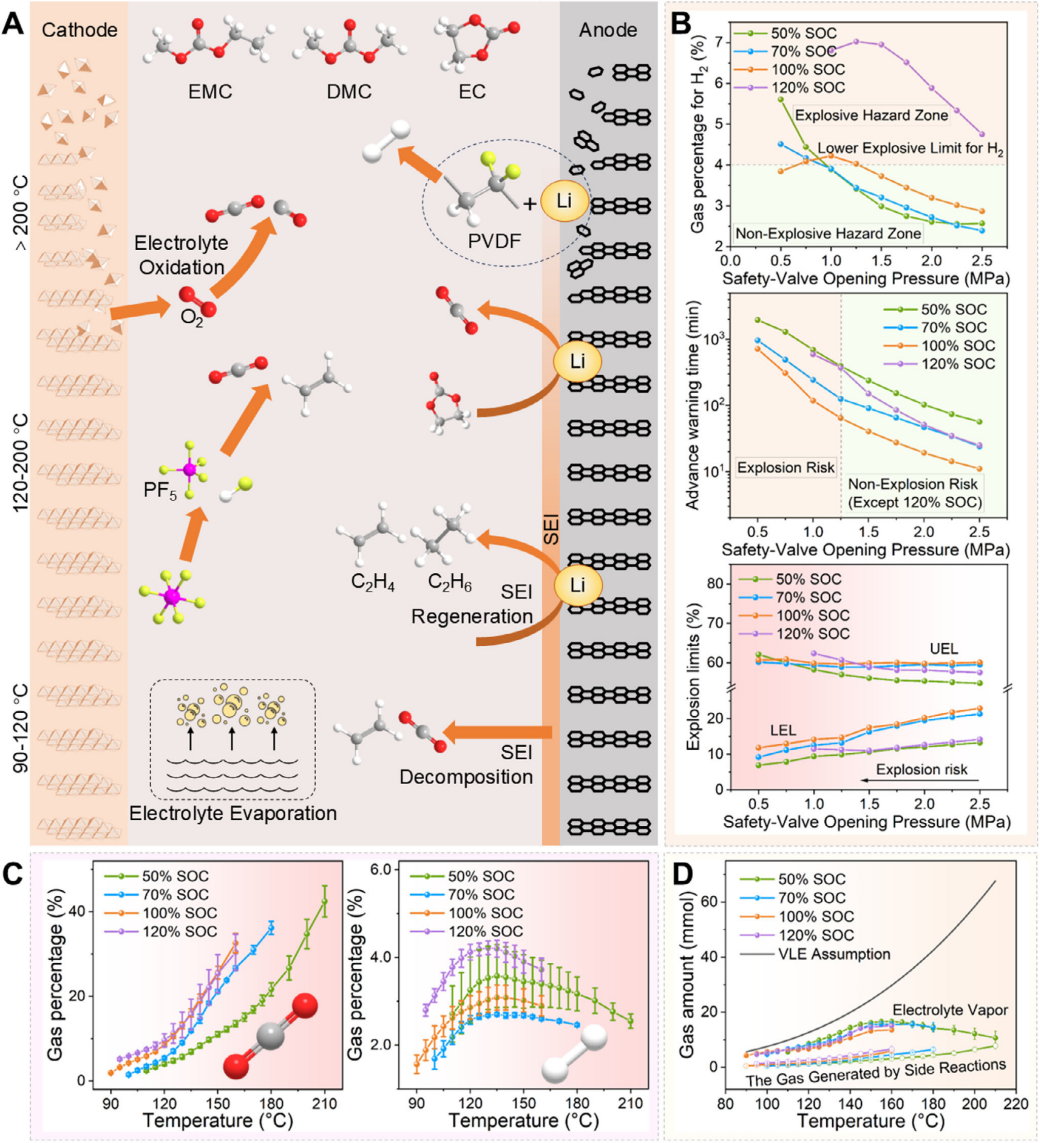
Gas generation behavior in LIBs during the HA stage. Data points represent the mean values of three independent replicates, sample size n=3; error bars indicate the experimental error. (A) Schematic illustration of gas generation pathways in LIBs during the HA stage. The electrolyte undergoes more intense evaporation while simultaneously participating in the chemical reaction. (B) Effects of safety‐valve opening pressure on advance warning time and explosion risk of vented gases. (C) Temperature‐dependent evolution of gas components during the HA stage for LIBs at 50%/70%/100%/120% SOC. Gas samples were collected starting from *T*_onset_ with 5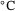 intervals, and increased to 10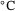 intervals when approaching the violent runaway stage. (D) Temperature‐dependent variations in electrolyte vapor and gases generated by side reactions during the HA stage.

The evolution of cell temperature and internal vessel pressure with various SOCs is illustrated in Figure S3. The results indicate that the battery temperature continues to rise due to the chemical reactions. With increasing SOC, the duration of the HA stage is markedly shortened, while the TR trigger temperature decreases and the peak temperature during TR increases, indicating an elevated risk and severity of TR at higher SOCs. These SOC‐dependent differences in thermal and pressure evolution reflect variations in heat release behavior, which subsequently govern the gas generation characteristics. The evolution of gas components with temperature during the HA stage for cells with varied SOCs is depicted in Figure 3C and Figure S6. The temperature‐dependent gas generation rates for HA stage are further provided in Figure S13. Across all SOC levels, CO_2_ is the predominant gas generated, reaching concentrations exceeding 30% by the end of the HA stage. Following CO_2_, H_2_ and CO are the next most prevalent gases, with peak concentrations ranging from 4% to 6%. In comparison, CH_4_ and C_2_H_4_ remain at around 1%, while C_2_H_6_ is the least abundant component, with a maximum concentration of about 0.2%. At temperatures below 125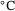, the concentration of gaseous species exhibits a positive dependence on the SOC. An exception is observed for H_2_, where its proportion at 50% SOC exceeds that at 70% and 100% SOC, reflecting the nonlinear dependence of HF‐related reactions on SOC. At elevated temperatures exceeding 125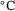, cells with 50% SOC exhibit higher generation ratios of several gaseous species, including CO, CH_4_, C_2_H_4_, and C_2_H_6_, compared to higher SOC conditions. This behavior can be attributed to the slower heat generation rate at 50% SOC, which prolongs the reaction duration, as well as the higher separator collapsing temperature, *T*_sc_, allowing gas generation pathways to proceed more completely during the later HA stage. Additionally, as shown in Figure S13b, the H_2_ generation rate stops decreasing at approximately 120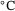 and begins to increase as the temperature rises further. This indicates that internal side reactions start to contribute to H_2_ production after 120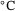.

During the HA stage of commercial batteries, the generated gas accumulates within the cell until the internal pressure triggers the opening of the safety valve. If the vented gas falls within their explosion limit, ignition or explosion may occur upon exposure to an ignition source. Moreover, the timing of safety valve opening is closely coupled with the activation of the gas alarm in the BESS, making the safety‐valve design a critical factor in early warning and hazard mitigation. Based on the experimental results, the explosion risk of the vented gas and the advanced warning time with different safety‐valve opening pressures are analyzed and summarized in Figure 3B. N_2_ is excluded from the analysis, with the other gases re‐normalized. Focusing on H_2_, reduced safety‐valve opening pressures extend the advanced warning time. However, it also increases the likelihood that the H_2_ concentration in the vented gas exceeds its 4% lower explosion limit (LEL). When the safety‐valve opening pressure is designed to be above 1.25 MPa, the vented gases from cells at 50%–100% SOC remain below the explosion threshold for H_2_. It should be noted that the vented gas is a multicomponent combustible mixture rather than pure H_2_. Therefore, the LEL and upper explosion limit (UEL) of the mixed vented gases are calculated using Equation S11, and the results show that lower safety‐valve opening pressures indeed correlate with higher explosion risks. Consequently, battery safety design should balance the trade‐off between maximizing early warning time and minimizing the explosion risk of vented gases.

Moreover, electrolyte vapor constitutes a major contribution to gas generation in LIBs throughout the HA stage. Figure 3D presents the temperature‐dependent evolution of electrolyte vapor and gases generated from side reactions. When interpreted in conjunction with Figure 2C, it is evident that during the CA stage and the early HA stage, electrolyte volatilization remains largely evaporation‐dominated and broadly consistent with VLE behavior, while chemical consumption plays a secondary role. However, in the later stage of HA, particularly when the cell temperature exceeds 150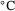, the electrolyte vapor amount becomes progressively lower than that predicted by VLE assumption. This increasing reduction demonstrates that the residual electrolyte vapor inside the cell participates in the reactions. In addition, the consumption of the liquid electrolyte by chemical reactions disrupts the local vapor‐liquid dynamic equilibrium, which either drives the vapor to condense or suppresses further evaporation. Consequently, the reduction in both phases leads to a breakdown of the conventional VLE‐based description that is widely adopted in the literature [69, 70].

Following a violent runaway stage, large quantities of flammable gases are generated, which critically influence post‐TR fire behavior. As shown in Figure S4, CO_2_ and CO dominate the post‐TR gas composition across all SOCs, together accounting for approximately 60%–67%. For cells with SOCs below 100%, CO_2_ is more abundant, reaching 58% at 50% SOC, while CO is correspondingly lower (about 9%), reflecting more complete oxidation associated with greater residual electrolyte. H_2_ represents the second major component (21%–26%), whereas C_2_ hydrocarbons remain below 4% but can still affect the explosion limits of the vented gas mixture [71].

### 2.3 Development of GGKNet for Gas Evolution

Based on the above experimental observations, a wide spectrum of gaseous species, including H_2_, CO, CO_2_, C_1_‐C_2_ hydrocarbons, and electrolyte vapor, is continuously generated during the CA and HA stages. The corresponding reaction pathways are intrinsically complex, as partially summarized in Tables 1 and 2, which compile the gas generation mechanisms that have been identified and discussed in previous studies. However, the experimentally observed gas generation rates exhibit pronounced nonlinear dependencies on both temperature and SOC, indicating that, beyond the established pathways, additional elementary reactions are likely involved.

In principle, Arrhenius‐type formulations provide a classical framework for describing chemical reaction kinetics of a specific reaction system. Nevertheless, when dozens of involved reactions are simultaneously considered, the number of unknown kinetic parameters, including stoichiometric coefficients, pre‐exponential factors A, activation energies $E_{a}$, and temperature exponents b, rapidly reaches the order of $O(10^{3})$. Moreover, gas evolution is further coupled with multiple physicochemical processes, such as gas adsorption and desorption on the porous graphite anode, evaporation and partial consumption of liquid electrolytes, and chemical processes involving lithium. These high‐dimensional parameter spaces and strong multi‐physics couplings make accurate and robust prediction of gas generation during the CA and HA stages extremely challenging, not only for conventional kinetic modeling approaches, but also for existing data‐driven and neural‐network‐based methods that lack explicit physical interpretability and reaction‐level constraints.

To address this complexity, the GGKNet framework is proposed as shown in Figure 4. Figure 4A illustrates the overall architecture, which integrates a gas‐generation chemical reaction network (CRN) with three physically constrained sub‐models to capture the coupled multi‐physics governing gas evolution during TR. Specifically, during TR, the distribution of Li among the cathode, anode, and electrolyte evolves dynamically. To account for uncertainty in lithium availability, an effective lithium inventory ${\mathrm{Li}}_{\mathrm{eff}}$, is introduced and dynamically adjusted through a dedicated Li state model (LSM). The LSM consists of a main residual network with two residual blocks, which takes temperature, T, and ${\mathrm{Li}}_{\mathrm{SOC}}$, which is the lithium content in the battery associated with SOC, as inputs to predict corrections, complemented by a lightweight auxiliary network to improve SOC discriminability. This coordinated design allows the LSM to capture temperature‐ and SOC‐dependent Li redistribution across the system, enabling consistent coupling between chemical processes and gas‐generation kinetics while mitigating overfitting and preserving sensitivity to different SOCs. In addition to LSM, GGKNet incorporates two other key physical processes that influence gas evolution. Specifically, an electrolyte evaporation sub‐model describes the temperature‐driven phase transition of liquid electrolyte into the gas phase, while an adsorption–desorption sub‐model captures the reversible uptake and release of H_2_ on the porous graphite anode. Note that, the evaporated electrolyte is further allowed to participate in subsequent side reactions, enabling coupled treatment of phase transition and chemical consumption. By embedding these three sub‐models within a unified learning framework of reaction kinetics, GGKNet constrains data‐driven predictions with essential chemical and transport processes, thereby improving physical consistency, numerical stability, and predictive robustness under limited experimental data availability.

**FIGURE 4:**
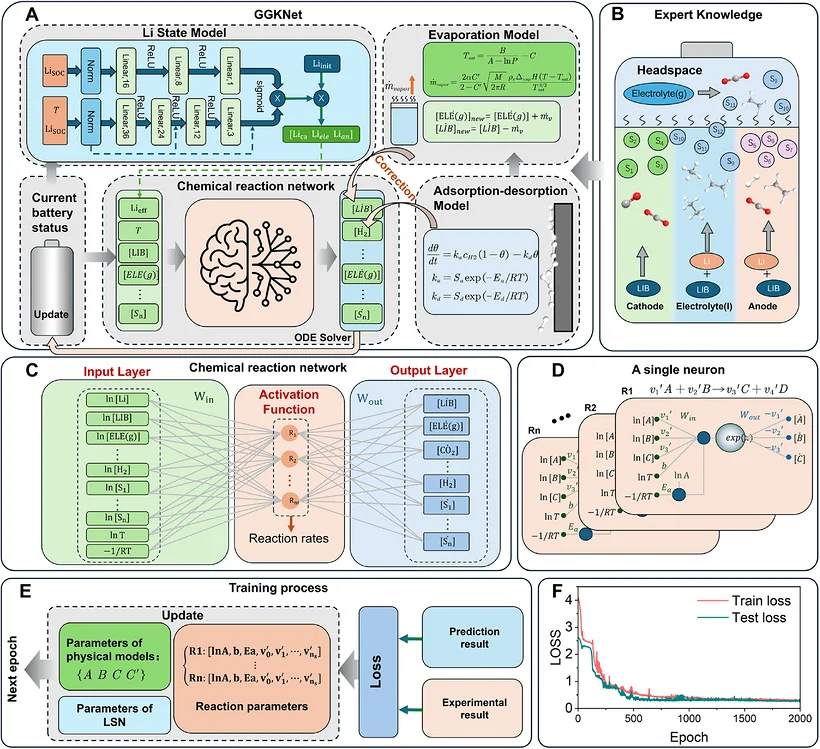
Schematic illustration of the construction of the gas generation kinetics network (GGKNet). (A) Overall architecture of GGKNet, integrating a gas‐generation chemical reaction network (CRN) with three physically constrained sub‐models, including the Li state model (LSM), the electrolyte evaporation model, and the gas adsorption‐desorption model. (B) Expert‐knowledge‐based partitioning of the CRN into four reaction domains. (C) Schematic of the CRN embedded within GGKNet. (D) Neuron‐level representation of an individual reaction within the CRN. (E) Training processes of the GGKNet. (F) Representative training and validation loss curves during model training.

Within GGKNet, the gas generation kinetics are learned through a CRN. To enhance the learning efficiency, some gas generation kinetics expert knowledge extracted from Tables 1 and 2 is pre‐embedded into the CRN. The CRN will automatically explore unknown reaction pathways and predict the corresponding kinetic parameters during training. As shown in Figure 4B, side chemical reactions occur at distinct locations within the cell. To reflect this spatial heterogeneity, the cell is partitioned into four reaction domains, namely the cathode domain, the anode domain, the liquid electrolyte domain, and the gaseous electrolyte domain. Established gas‐generation reactions are pre‐assigned to these domains, thereby restricting each reaction to physically plausible locations. In addition, to prevent the limitations of this expert knowledge from restricting the predictive capability of GGKNet, additional reactions have been incorporated into each domain for GGKNet to learn autonomously. The number of additional reactions significantly influences the stiffness of GGKNet. An insufficient number may lead to training oscillations, whereas an excessive number can easily cause overfitting. Therefore, through pre‐training, this study adopts a balanced setting. For details, refer to page S10 and Figure S14 of Supplementary Materials. Moreover, to account for the limited mass transport of solid intermediates, each domain is assigned a dedicated set of intermediate species ($S_{1}$‐$S_{n}$). At each time step, the LSM first updates ${\mathrm{Li}}_{\mathrm{eff}}$ in the cathode, anode, and electrolyte. The Li states are then incorporated into the input vector of the CRN, which predicts species‐wise mass change rates arising solely from chemical reactions. These rates are subsequently updated by the electrolyte evaporation network and the H_2_ adsorption‐desorption network to account for non‐reactive physical processes.

Figure 4C,D illustrates the detailed structure of the CRN embedded within GGKNet and the architecture of a single neuron, respectively. As shown in Figure 4D, a single neuron represents an individual chemical reaction, e.g. 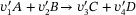. The inputs are the instantaneous concentrations of the species and the local temperature, while the outputs are the reaction rate and the stoichiometric coefficients of the reactants and products. The neural parameters encode the activation energy, $E_{a}$, pre‐exponential factor, A, temperature exponents, b, and the stoichiometric coefficients, $v_{1}^{\prime }-v_{4}^{\prime }$, allowing computation of reaction rates consistent with the Arrhenius equation. This structure ensures that each neuron is physically interpretable, producing reaction rates that follow established chemical kinetics, while allowing data‐driven optimization to capture nonlinear dependencies on temperature and SOCs. By stacking multiple single neurons, a complete CRN is constructed, as shown in Figure 4C. The input layer receives a vector composed of ${\mathrm{Li}}_{\mathrm{eff}}$, battery active materials (LIB), generated gases (CO_2_, CO, ELE(g), C_2_H_4_, C_2_H_6_, H_2_, CH_4_), other unknown intermediate species ($S_{1}$‐$S_{n}$), and temperature‐related terms. Notably, ${\mathrm{Li}}_{\mathrm{eff}}$, H_2_, and electrolyte evaporation and consumption are influenced by the three sub‐models illustrated in Figure 4A. The output reaction rates from all neurons are then aggregated to determine the net production and consumption rates of all species, forming a coupled system of ordinary differential equations (ODEs) that govern the temporal evolution of chemical composition. Numerical integration of these ODEs updates the state of the system, which is subsequently fed back as input to the network at the next step, enabling dynamic, time‐resolved prediction of gas generation.

The training process is further illustrated in Figure 4E. In each training epoch, GGKNet predicts the temporal evolution of gas mass by numerically integrating the coupled ODEs defined by the CRN considering the associated physical sub‐models. The predicted gas species profiles are then compared with the corresponding experimental data to compute the loss function (Equation 8). The network parameters are then updated using the Adam optimizer, a stochastic gradient‐based optimization algorithm that combines momentum with adaptive learning rates to ensure efficient and numerical stable convergence [72]. In addition to the chemical kinetic parameters in the CRN, the parameters of the LSM, the electrolyte evaporation model, and the gas adsorption‐desorption model are jointly updated during training. Further implementation details are provided in the Methods section. After 2000 epochs of training, as shown in Figure 4F, the training demonstrates good convergence without overfitting.

Figure 5 summarizes the training outcomes of GGKNet, where the gas generation chemical reaction pathways learned by GGKNet (denoted as learned reactions, LRs) during the HA and CA stages are illustrated in Figure 5A. Notably, the newly identified LRs inferred by GGKNet exhibit strong consistency with established gas‐generation mechanisms reported in the literature, indicating that the model is capable of recovering physically meaningful chemistry beyond predefined reactions. In the anode domain, LIB reacts with Li to generate CO_2_ through LR8, 10, and 12, which may correspond to SEI decomposing reactions (Reactions 4–7). The SEI regeneration reaction producing C_2_H_4_ (Reactions 12–14) is captured by LR9. Moreover, LR14 can yield CO and H_2_, which can correspond to Reactions 2, 3, and 10. Beyond direct gas formation involving LIB, these reactions generate intermediate species that further participate in secondary pathways. For instance, LR9 produces $S_{6}$‐$S_{8}$, whereas LR10‐12 generate $S_{5}$‐$S_{8}$ and $S_{9}$‐$S_{12}$. Among them, $S_{5}$‐$S_{8}$ can further react with $S_{10}$‐$S_{11}$ in the presence of Li via LR16 to produce $S_{12}$, which subsequently reacts with Li through LR15 to generate CO_2_ and CH_4_. In the cathode domain, LIB reacts with Li through LR2 to produce CO and intermediate species $S_{1}$‐$S_{4}$, $S_{9}$‐$S_{12}$, corresponding to Reactions 25 and 26. These intermediate species can also be generated by LR3. Among them, $S_{1}$‐$S_{4}$ can react with $S_{10}$‐$S_{11}$ and LIB with the participation of Li to generate $S_{9}$ and $S_{12}$ in LR5. Subsequent reactions of $S_{12}$ with Li (LR6‐7) produce CH_4_, C_2_H_6_, C_2_H_4_, CO_2_, CO, and enable the intermediate species $S_{1}$‐$S_{4}$ and $S_{10}$‐$S_{11}$ to regenerate. This reaction loop corresponds to the interaction between the cathode electrode and the electrolyte. In the liquid electrolyte domain, LIB and Li generate C_2_H_6_ through LR19, corresponding to Reaction 15, and generate C_2_H_4_ via LR17, consistent with Reactions 13 and 14. Among various intermediate species, $S_{12}$ plays a central role in this domain, participating in multiple reaction pathways. Specifically, $S_{12}$ is formed through LR17‐LR22 and subsequently reacts with Li via various pathways (LR21‐LR28) to generate CH_4_, C_2_H_6_, C_2_H_4_, CO, and CO_2_. In addition to chemical reactions, the liquid electrolyte can evaporate into the gaseous electrolyte phase (ELE(g)) and enter the headspace. In this domain, ELE(g) directly produces C_2_H_4_ via LR29 and generates $S_{9}$‐$S_{12}$ through LR29‐31. Among these intermediate species, $S_{12}$ can be converted into $S_{9}$‐$S_{11}$ via LR33‐36. Additionally, $S_{12}$ can also further decompose into CO and CO_2_ (LR33‐36), as well as CH_4_, C_2_H_6_, and C_2_H_4_ (LR33, LR35). These reaction pathways correspond to the oxidative decomposition reactions of the electrolyte (Reactions 23–26). The complete chemical reaction mechanism learned by GGKNet is detailed in Table S5.

**FIGURE 5:**
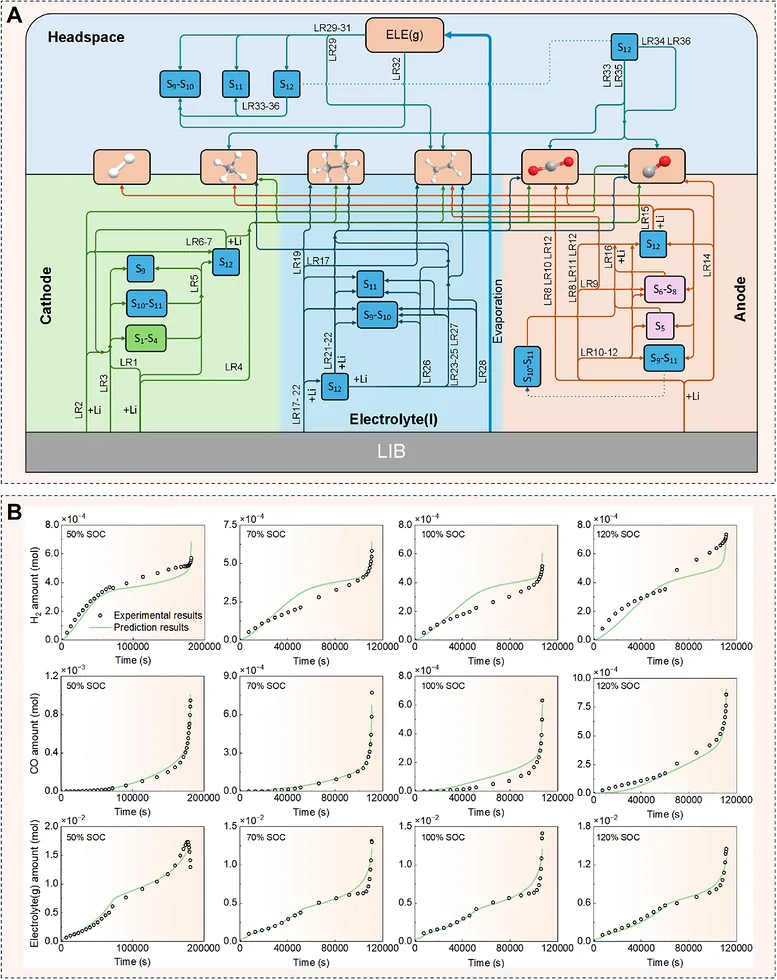
Learned gas generation reaction pathway and predictive performance of GGKNet. (A) Previously unknown gas generation reaction pathway of HA and CA stages explored by GGKNet. (The dashed line between the two $S_{12}$ blocks indicate that they represent the same substance.) (B) Comparison between GGKNet‐predicted and experimentally measured gas evolution for representative species.

Figure 5B compares the GGKNet predictions with experimental results for H_2_, CO, and ELE(g) under different SOC conditions, while results for other gas species are detailed in Figure S7. Overall, GGKNet demonstrates high predictive accuracy across a wide range of SOCs and for multiple gas components, indicating that the learned reaction pathways capture the dominant gas‐generation mechanisms. In particular, the generation rate of H_2_ exhibits a pronounced nonlinear dependence on temperature during the CA stage, rather than following a simple Arrhenius‐type trend. GGKNet accurately reproduces this behavior owing to the incorporation of the adsorption‐desorption model, which explicitly accounts for the reversible uptake and release of H_2_ on the graphite anode. On the contrary, if this model is not considered in GGKNet, the predictive performance for H_2_ significantly decreases (see Figure S11). For ELE(g), GGKNet successfully captures the distinct generation behaviors during the CA and HA stages and correctly predicts the consumption of ELE(g) toward the end of the HA stage at lower‐SOC conditions. This behavior arises from two coupled mechanisms explicitly represented in GGKNet, namely temperature‐driven electrolyte evaporation and subsequent chemical consumption of the evaporated species. The evaporation sub‐model constrains the phase transition of ELE(l), while the reaction network allows ELE(g) to participate in downstream reactions, together enabling accurate prediction of ELE(g) evolution across different SOCs and thermal stages. Meanwhile, the electrolyte evaporation model also enables the subsequent three‐dimensional model to accurately predict the pressure in the headspace, thereby precisely determining the safety‐valve opening time (Section 2.4). Therefore, it is crucial to establish an electrolyte evaporation model. The dataset used for training the GGKNet comes from experimental data, encompassing diverse SOC conditions ranging from 50% to 120% and a broad temperature range spanning from 50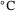 to above 200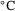. For each SOC condition, 20–30 data points are utilized, with each data point including temperature data as input and concentration data for seven specific gases (H_2_, CH_4_, C_2_H_6_, C_2_H_4_, CO_2_, CO, and electrolyte vapor) as labels. Note that, despite the limited size of the training dataset, no overfitting is observed for any gas predictions on the test dataset (100% SOC). This robustness is attributed to the physics‐informed structure of GGKNet, which enforces physicochemical constraints, enhances interpretability, and substantially reduces the risk of overfitting.

### 2.4 Experimental Validation of GGKNet

The reaction mechanism constructed by GGKNet requires validation under realistic TR conditions, and Figure 6 presents the related experimental and simulation methods together with the validation results. To this end, a dedicated thermal‐abuse TR experiment is designed to mimic TR scenarios in BESS within a confined environment. Specifically, TR of commercial cells is triggered in a constant volume combustion chamber (CVCC), as shown in Figure 6A. This configuration enables controlled reproduction of the key stages relevant to BESS safety, namely pre‐vent pressure buildup, safety‐valve opening, transient gas venting and dissipation with early warning by gas sensors, and following combustion or explosion in the enclosure. The time‐resolved evolution of pressure, temperature, and gas composition is simultaneously measured using pressure transducers, thermocouples, and gas sensors. Note that, the gas signals of H_2_ and CO are selected as they are employed as primary detection targets for early warning systems in commercial BESSs [15]. In parallel, a high‐speed Schlieren diagnostics system is employed to visualize the gas venting and subsequent combustion processes. The TR characteristics of the cells within the CVCC exhibit good repeatability across two independent experiments, as shown in Figure S9. A detailed schematic of the experimental setup is provided in Figure S8, while specifications of the test cell, the CVCC, Schlieren system, and time‐resolved sensing system are summarized in Tables S1–S4, respectively.

**FIGURE 6:**
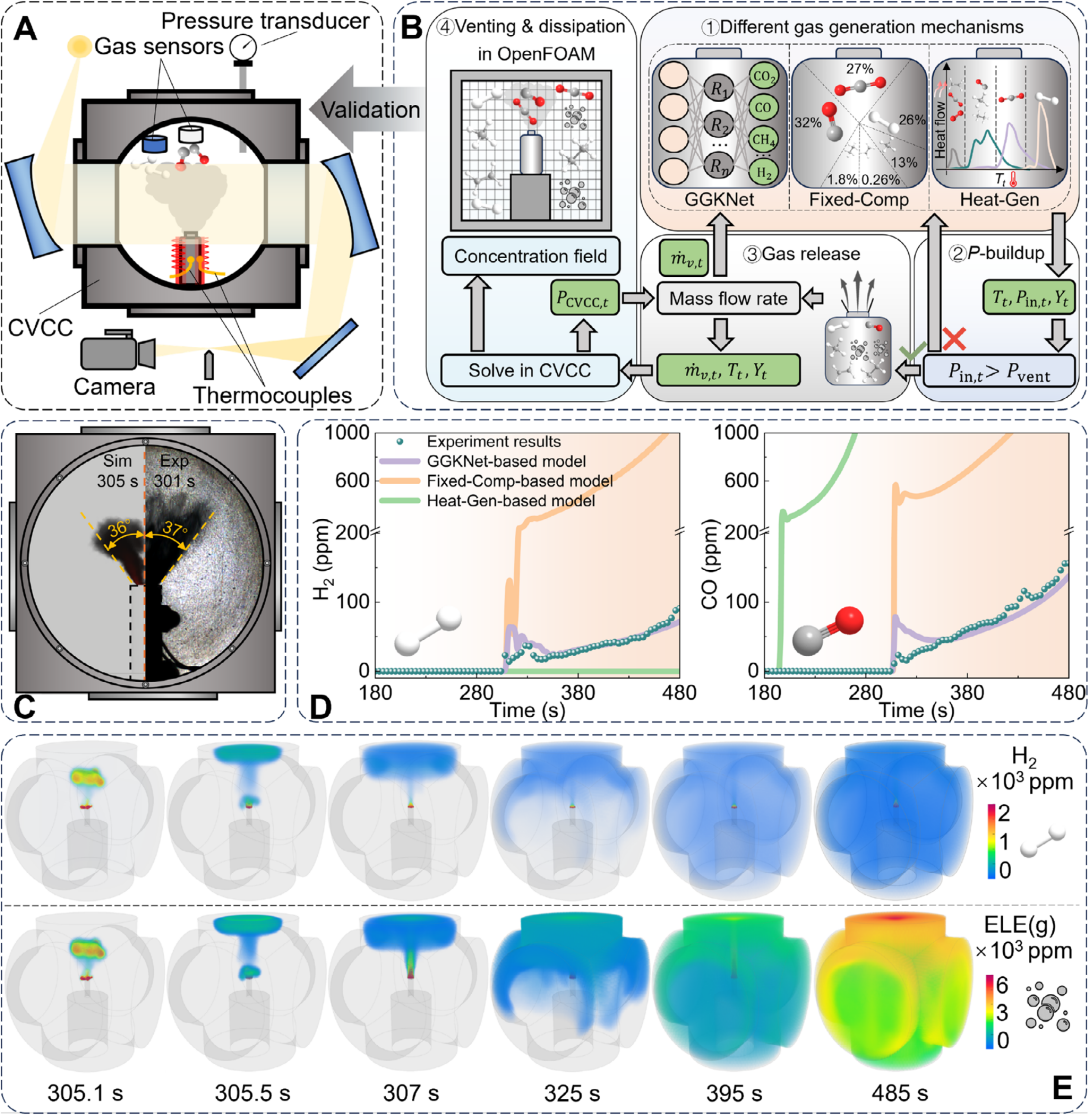
Experimental validation of GGKNet for pressure buildup, transient venting and early‐warning‐relevant metrics during TR. (A) Schematic of multidimensional sensing for gas‐based early warning during thermal‐abuse TR experiment in confined environments. (B) Schematic of three‐dimensional gas venting and dissipation CFD simulation model coupling different gas generation mechanisms. (C) Comparison of venting onset and angle between simulation results from the GGKNet‐based model and Schlieren images. (D) Temporal evolution of H_2_ and CO concentration in experiments and simulation results with different gas generation mechanisms. (E) Spatiotemporal concentration contours of H_2_ and ELE(g) inside CVCC from GGKNet‐based model.

To evaluate the predictive capability of different gas generation mechanisms, the GGKNet‐based mechanism and two representative conventional mechanisms are coupled with an identical gas release model and three‐dimensional CFD framework in OpenFOAM [73], using the same battery temperature input to simulate the HA process, as illustrated in Figure 6B. The conventional approaches include (i) a fixed‐composition‐based (Fixed‐Comp) model [74], in which the vented gas composition is prescribed a priori, and (ii) a heat‐generation‐based (Heat‐Gen) model [37], where gas release is inferred from the heat generation rate. By contrast, GGKNet directly predicts the transient mass generation of individual gas species based on the learned reaction mechanism, as shown in Figure 5. To ensure a fair comparison, the three models adopt nearly identical boundary conditions (pages S8–S10), including the battery surface temperature based on experimental conditions and the temperature of CVCC walls set at 25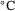. The differences among the models are reflected only in the prediction of gas generation. A co‐simulation framework is constructed to reproduce the experiments, with the computational workflow summarized in Figure 6B. At each time, t, GGKNet calculates the gas generation from the reaction rates of individual pathways at the battery temperature, $T_{t}$, from which the internal pressure, $P_{\text{in},t}$, and the mass fraction of gas components, $Y_{t}$, are obtained by solving the governing ODE equations. $P_{\text{in},t}$ is then compared with the venting threshold, $P_{\text{vent}}$, of the battery safety valve [30]. Once $P_{\text{in},t}$ exceeds $P_{\text{vent}}$, gas release is triggered and the resulting mass flow rate, ${\dot{m}}_{v,t}$, is imposed to OpenFOAM, with $Y_{t}$ and $T_{t}$ supplied as boundary conditions to resolve the transient flow and species transport within the CVCC.

The chamber pressure, $P_{\text{CVCC},t}$, computed by OpenFOAM is subsequently fed back to the gas release model for the next time step, while ${\dot{m}}_{v,t}$ is returned to GGKNet to update gas mass balances. This iterative coupling is repeated throughout the transient venting simulation.

Figure 6C first evaluates the pressure buildup and initial venting behavior predicted by the GGKNet‐based model with Schlieren observations. During the heating stages, gases generated inside the cell lead to a gradual accumulation of internal pressure, which is consistently captured by the model. The experimentally observed safety‐valve opening occurs at 301 s, while GGKNet predicts venting onset at 305 s, validating the accuracy of the modeled pressure buildup process. Beyond the venting onset, the jet morphology and venting angle calculated by the model align closely with the Schlieren image, indicating a physically consistent description of the gas release dynamics. The safety valve opening triggers rapid release of accumulated gases from CA and HA stages, resulting in a sharp increase of vented‐gas concentration above the cell. Figure 6D compares predicted H_2_ and CO concentrations with experimental measurements. Overall, the GGKNet‐baesd CFD model demonstrates the best agreement with the gas‐sensor measurements inside CVCC, demonstrating its ability to accurately capture gas generation, venting, and subsequent dissipation. In contrast, the Fixed‐Comp model cannot resolve in‐cell pressure buildup and therefore requires the venting time to be prescribed using the GGKNet prediction. Even so, it overestimates the concentrations of H_2_ and CO, particularly during the early venting stage, because it assumes a fixed final gas composition that neglects the transient evolution of gas generation prior to safety valve opening. Moreover, the Heat‐Gen model assumes a linear correlation between heat and gas generation, leading to an overestimation of gas evolution during the CA and HA stages. Consequently, the predicted safety valve opening time (195 s) occurs significantly earlier than the experimental result, resulting in overly optimistic estimates for early warning by gas signal of CO generated by low‐temperature anode reactions in the assumption. Furthermore, in this model, the earliest heat generation reaction, the SEI layer decomposition, is assumed to solely generate C_2_H_4_, CO_2_, and O_2_, neglecting H_2_ generation from Reactions 2‐3 during the CA stage before this process. As a result, H_2_ generation is restricted to the reaction between the PVDF binder and Li (Reaction 27) and is therefore activated only during the violent runaway stage, failing to capture the early H_2_ evolution. Moreover, neither the Fixed‐comp model nor the Heat‐Gen model can characterize the generation and spatial distribution of ELE(g) within or outside the cell, despite the fact that such information is highly relevant for early‐warning applications.

The concentration distributions of H_2_ and ELE(g) are further visualized to support gas‐based early warning analysis, as illustrated in Figure 6E. Immediately after the safety‐valve opening, both species form a transient high‐concentration zone near the safety valve and are transported upward, dominated by the strong initial venting momentum. Moreover, the vent impingement of the chamber walls rapidly induces flow deflection, vortex formation, and lateral spreading, accompanied by a brief upstream reflux near the vent due to the high venting velocity. As the internal cell pressure and venting velocity decays, the venting flow transitions from a momentum‐dominated jet to a buoyancy‐driven plume, and gas transport becomes increasingly governed by molecular diffusion. Consequently, the initially stratified gas layers gradually evolve into a broader spatial distribution within the confined space. Beyond their distinct venting amounts, H_2_ and ELE(g) exhibit markedly different transport behaviors. Owing to its low density and high diffusivity, H_2_ preferentially accumulates near the top of the chamber and rapidly evolves toward a spatially uniform distribution. In contrast, ELE(g), with higher density and lower diffusivity, remains more localized, persisting in low‐velocity regions such as the upper corners, following being temporarily lifted by jet momentum. These results demonstrate that the GGKNet‐based CFD framework can explicitly resolve the spatiotemporal evolution of multiple gas species during TR, providing physics‐based guidance for gas‐sensor design, detection limits, and measurement strategies in BESSs.

## 3 Discussion

Thermal runaway in LIBs has conventionally been interpreted as a predominantly thermally driven failure process, in which HA is regarded as the earliest observable indicator of instability. This thermally centered interpretation has fundamentally constrained early‐warning strategies, as monitored thermal, electrical, and mechanical responses only manifest after the battery has crossed into an irreversible failure regime. In this work, we demonstrate that this limitation originates from an incomplete understanding of the earliest stages of battery degradation. By introducing operando gas monitoring as a chemically sensitive diagnostic, we show that the gas generation pathway is initiated well before measurable HA occurs. Specifically, we identify a previously unrecognized “CA” stage that precedes the HA stage, during which coupled electrolyte–electrode reactions are activated at temperatures as low as ∼ 60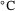. Although no discernible temperature rise is observed during this stage, trace gaseous species, dominated by CO_2_ and H_2_, begin to accumulate continuously, progressively increasing the susceptibility of the cell to subsequent thermal runaway. These results demonstrate that gas evolution, rather than temperature rise, constitutes the earliest experimentally observable signature of impending failure, thereby shifting the basis of early warning from a purely thermal criterion to a chemically activated one. Moreover, the dynamic warning thresholds for the internal gas sensors should be dependent on temperature and SOCs. By summarizing the gas production patterns during the CA and HA stages, this study proposes that the design of the safety valve opening pressure should balance the trade‐off between maximizing early warning time by external gas sensors and minimizing the explosion risk of vented gases.

Building on the identification of the CA and HA stages, this work further establishes a mechanistically grounded framework to uncover and quantify early‐stage gas generation during thermal runaway. By integrating operando gas measurements with a physics‐informed gas generation kinetics network (GGKNet), we enable the autonomous exploration of previously unresolved reaction pathways underlying gas evolution, rather than prescribing them a priori. GGKNet explicitly couples chemical reaction kinetics with gas adsorption‐desorption, electrolyte evaporation, and lithium‐involved reactions, allowing the origins and formation sequences of individual gas species to be resolved within a unified kinetic framework. As a result, the generation and accumulation behaviors of H_2_, CH_4_, CO, CO_2_, C_2_H_4_, C_2_H_6_, and electrolyte vapor, are quantitatively captured across a wide range of SOCs, revealing distinct, SOC‐dependent evolution pathways during the CA and HA stages. Beyond predictive accuracy, the kinetics‐network‐based design establishes a transparent, physically interpretable link between observed gas signals and their underlying physicochemical processes, enabling model outputs to be translated into actionable safety insights across operating conditions and cell states. The kinetic parameters learned by GGKNet therefore provide mechanism‐consistent guidance for gas‐based early‐warning technologies, informing target gas selection, alarm threshold definition, and warning timing for both internal and external sensing.

To assess its engineering relevance, the proposed framework is validated under realistic thermal runaway scenarios, where the temporal evolution of key gaseous species (H_2_ and CO) is experimentally characterized in large‐volume environments relevant to practical battery systems and early‐warning deployment. By coupling the GGKNet‐resolved gas generation mechanism within a three‐dimensional computational fluid dynamics (CFD) model, the framework captures both the in‐cell pressure buildup behavior and the subsequent species‐resolved concentration evolution following venting, enabling a consistent description of gas generation, release, and dissipation. Compared with existing models, the proposed framework demonstrates substantially improved performance in predicting the onset timing, generation characteristics, and spatial distribution of various gas species. Based on the CFD simulation results, the distribution of the vented gases in the space is non‐uniform and depends on the gas species. Therefore, for the external gas sensors, the setting of warning threshold should comprehensively consider the enclosure volume, the sensor installation location, and the zero‐point drift caused by long‐term operation. These results confirm that embedding gas generation kinetics into engineering‐scale modeling provides a robust and deployable foundation for gas‐based early‐warning strategies, advancing battery safety design from reactive thermal monitoring toward chemically informed preemptive intervention.

## 4 Materials and Methods

### 4.1 Preparation of the Cell Samples

Commercial 18650‐type LiNi_0.8_Co_0.1_Mn_0.1_O_2_ (NCM811) cells (Shenzhen Doublepow Technology) from the same production batch with detailed parameters listed in Table S1 were tested. Pristine cells were first fully charged to rate their capacity by applying a 1 A constant current until reaching a cutoff voltage of 4.2 V, followed by a constant‐voltage charge until the current decayed to below 40 mA. Following a waiting period of 60 min, the cells were discharged at 1 A until they reached the cut‐off voltage of 2.5 V. Each cell underwent five complete charge–discharge cycles to accurately rate its capacity, with the relative capacity errors found to be within 2%, after which the cells were recharged to target states of charge (SOCs) based on the ratio of charged capacity to rated capacity. After the charging process, a stabilization period of 24 h was ensured for the cell before the thermal abuse experiment in accelerating rate calorimeter or constant volume combustion chamber. Four SOC levels were selected in this study, namely 120%, 100%, 70%, and 50%, respectively, with 120% representing an overcharged condition that may trigger TR through electrical abuse.

### 4.2 Operando Gas Diagnostics During TR

The operando gas diagnostics platform consisted of three components, namely an accelerating rate calorimeter, an operando gas sampling system, and a gas chromatography diagnostic system. The ARC (BAC‐90A, Hangzhou YOUNG Instruction Science & Technology Co., Ltd.) was used to provide controlled thermal abuse conditions, while gas species were analyzed using a micro gas chromatograph (GC, Agilent 990 Micro GC, Agilent Technologies Inc.). During testing, the cell was placed in a well‐sealed stainless‐steel vessel, with the safety valve of the test cell pre‐opened. A schematic illustration of the experimental setup is depicted in Figure S1. Thermal abuse experiments were conducted using the heat‐wait‐seek (H‐W‐S) protocol. The initial temperature was set to 50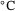, followed by temperature increments of 5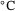. A self‐heating rate threshold of 0.01 K min^−1^ was used to identify the onset of exothermic reactions. The waiting time at each temperature step was fixed at 30 min to ensure thermal equilibration. During the H‐W‐S procedure, gas samples were periodically extracted from the vessel through the gas sampling system and subsequently analyzed by GC to determine gas composition without affecting thermal characteristics of cells in the ARC. A filter was installed at the outlet of the gas sampling line to prevent the loss of electrolyte vapor and condensable species. Note that the experiments during CA and HA stages were repeated three times. The detailed results of the thermal characteristic parameters are provided in Table S6.

### 4.3 Multidimensional Sensing for Early TR Warning

The multidimensional sensing platform consisted of three main systems, namely a constant volume combustion chamber (CVCC), an optical Schlieren diagnostic system, and a time‐resolved sensing system. The CVCC provided a confined environment representative of practical energy storage installations, enabling controlled initiation and evolution of TR, while the Schlieren system was employed to visualize gas venting, jet development, and subsequent flow dynamics. The sensing system provided real‐time measurements of gas composition (H_2_ and CO), as well as synchronized temperature and pressure signals. During testing, cells with 100% SOC were mounted inside the CVCC and subjected to external thermal abuse using a resistive heating film attached to the cell surface, operated at a fixed power of 38 W until the end of TR. The safety valve of the cell remained closed prior to venting, allowing pressure buildup and valve opening to occur naturally during TR. Type‐N thermocouples monitored both cell surface and heater temperatures, while a high‐frequency pressure transducer captured transient pressurization associated with gas venting and combustion. Gas composition within the CVCC was measured in real time using dedicated sensors for H_2_ and CO. Simultaneously, high‐speed Schlieren imaging captured density gradients, enabling visualization of high‐velocity gas jets and flow structures induced by venting and subsequent combustion. A schematic illustration of the experimental setup is detailed in Figure S8.

### 4.4 Construction of CRN

As shown in Figure 4D, for a example chemical reaction 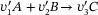. The reaction rate r of this chemical reaction can be expressed as:

(1) $$\begin{array}{cc} r & =AT^{b}\exp\left(-E_{a}/RT\right)A^{v_{1}^{\prime }}B^{v_{2}^{\prime }}C^{v_{3}^{\prime }}\\ & =\exp(v_{1}^{\prime }\ln\left[A\right]+v_{2}^{\prime }\ln\left[B\right]+v_{3}^{\prime }\ln\left[C\right]+b\ln T-\left(E_{a}/RT\right)+\ln A)\\ & =\exp(W_{\text{in}}x+B) \end{array}$$

where $v_{i}^{\prime }$ presents the stoichiometric coefficients of the reactants and products in this reaction. The $v_{i}^{\prime }$ values for reactants are positive, while those of products are negative. A refers to the pre‐exponential factor, $E_{a}$ is the activation energy of the chemical reaction, and b is the temperature correction coefficient. Based on Equation (1), the chemical reaction can be represented as a neuron in a CRN. Among these, ln[A], ln[B], ln[C], lnT, and −1/RT constitute the inputs of this neuron x, while $v_{i}^{\prime }$, b, and $E_{a}$ form the weights of the input layer $W_{\text{in}}$, with lnA serving as the bias B. Ultimately, the rate of mass change for each substance can be expressed as:

(2) $$\begin{array}{c} \frac{d[A]}{dt}=-v_{1}^{\prime }r\;\frac{d[B]}{dt}=-v_{2}^{\prime }r\;\frac{d[C]}{dt}=-v_{3}^{\prime }r \end{array}$$

It can be seen that $-v_{i}^{\prime }$ constitutes the weight $W_{\text{out}}$ of the output layer of the neuron, and the mass change rates of various substances are obtained at the output. By randomly initializing different weights and biases, neurons representing various chemical reactions can be obtained. Assembling a large number of these neurons ultimately forms the CRN as shown in Figure 4B.

### 4.5 Construction of GGKNet

In addition to the CRN model, GGKNet also includes three sub‐models. Among them, the evaporation rate ${\dot{m}}_{\text{vapor}}$ of the electrolyte can be obtained using Tanasawa's phase change model [37]:

(3) $${\dot{m}}_{\text{vapor}}=\frac{2\alpha C^{\prime }}{2-C^{\prime }}\sqrt{\frac{M}{2\pi R}}\frac{\rho_{v}\Delta_{\text{vap}}H\left(T_{t}-T_{\text{sat}}\right)}{T_{\text{sat}}^{3/2}}$$

In this equation, $C^{\prime }$ represents the evaporation coefficient of the electrolyte solvents, M is the molar mass, $\rho_{v}$ is the density of the electrolyte vapor, $\Delta_{\text{vap}}H$ is the latent heat of vaporization of the electrolyte. α is a correction factor that accounts for the effect of electrolyte consumption in the porous jelly roll on the evaporation rate [37], which decreases linearly with the consumption of LIB. $T_{\text{sat}}$ is the saturation temperature of the electrolyte, which can be calculated using the Antoine equation [75]:

(4) $$T_{\text{sat}}=\frac{B}{A-\ln P_{\text{ele}}}-C$$

where A, B, and C are parameters related to the characteristics of the electrolyte, and $P_{\text{ele}}$ represents the current partial pressure of the electrolyte vapor.

The adsorption and desorption processes of H_2_ are modeled using the Langmuir equation [76]:

(5) $$\frac{d\theta }{dt}=k_{a}c_{H_{2}}\left(1-\theta \right)-k_{d}\theta$$

The first and second terms of this equation correspond to the adsorption rate and desorption rate, respectively. Here, $c_{H_{2}}$ is the concentration of H_2_, and θ represents the H_2_ fractional coverage, defined as $\theta =m_{H_{2}}/m_{0}$. $m_{H_{2}}$ and $m_{0}$ denote the currently adsorbed mass of H_2_ and the maximum adsorbable mass of H_2_, respectively. $k_{a}$ and $k_{d}$ represent the temperature‐dependent adsorption and desorption rate constants, which can be calculated using the following equations [77]:

(6) $$k_{a}=S_{a}\exp\left(-E_{a}/RT\right)$$

(7) $$k_{d}=S_{d}\exp\left(-E_{d}/RT\right)$$

where $S_{a}$ and $S_{d}$ represent the frequency factors for adsorption and desorption, respectively, while $E_{a}$ and $E_{d}$ denote the energies for adsorption and desorption.

### 4.6 Model Training

The loss function employed uses the root mean square error (RMSE) as the loss metric, consisting of two terms. The first term measures the error between predicted and experimental gas mole values, while the second term penalizes mass non‐conservation during the integration process.

(8) $$\text{Loss}=\sum_{i}\sum_{t}\omega_{i}\text{RMSE}\left(\omega_{t}n_{i,t},\omega_{t}{\hat{n}}_{i,t}\right)+\sum_{t}\text{RMSE}\left(m_{\text{init}},\sum_{i}{\hat{m}}_{i,t}\right)$$

In the first term, $n_{i,t}$ and ${\hat{n}}_{i,t}$ represent the experimentally obtained and GGKNet‐predicted mole value of the gas component i at time t, respectively. By assigning different weights through $\omega_{t}$ to different time points, with weights linearly increasing as time progresses, GGKNet can more accurately predict the gas generation throughout the CA and HA stages. Moreover, considering the magnitude differences in the molar amounts of different gases, $\omega_{i}$ applies different weights to various gases, ensuring consistency in the magnitude of loss generated by different gases. The second term is the mass non‐conservation penalty term, where $m_{\text{init}}$ represents the initial mass of the species, and ${\hat{m}}_{i,t}$ denotes the GGKNet‐predicted mass of the gas component i at time t. Although mass conservation is ensured when constructing the reaction equations, the GGKNet parameters may deviate from reality during the training process, potentially accumulating significant numerical errors during integration, leading to mass non‐conservation and oscillations in the training process. Adding this term can help ensure the stability of the training process.

### 4.7 Model Validation

For the co‐simulation model used in model validation, this study primarily incorporates a gas release model to calculate the mass flow rate during the venting process, enabling bidirectional coupling between GGKNet and OpenFOAM. Specifically, given that $P_{\text{in},t}$ and $P_{\text{CVCC},t}$ are known, the gas venting velocity, $u_{t}$, can be calculated using the isentropic nozzle flow equation [78]:

(9) $$\begin{array}{cc} P_{v,t} & =\max\left(P_{\text{CVCC},t},\;{\left(\frac{2}{\gamma +1}\right)}^{\gamma /(\gamma -1)}P_{\text{in},t}\right),\\ Ma_{t} & =\min\left(1,\;\sqrt{\left({\left(\frac{P_{\text{in},t}}{P_{v,t}}\right)}^{(\gamma -1)/\gamma }-1\right)\frac{2}{\gamma -1}}\right),\\ u_{t} & =Ma_{t}\sqrt{\frac{\gamma P_{v,t}}{\rho }} \end{array}$$

where $P_{v,t}$ is the reference pressure at the nozzle orifice, γ is the heat capacity ratio of the vented gases, $Ma_{t}$ is the Mach number of the venting flow, $u_{t}$ is the gas venting velocity at the orifice exit, and ρ is the density of the vented gas. Considering that the under‐expanded venting flow will continue to expand after leaving the nozzle, the state parameters under steady‐state flow conditions should be obtained to serve as boundary conditions for subsequent three‐dimensional simulations. Therefore, the “pseudo‐diameter” is used to correct $u_{t}$ [79, 80]:

(10) $${\tilde{u}}_{t}=\left\{\begin{array}{cc} C_{D}u_{t}, & \text{subsonic flow}\\ C_{D}u_{t}-C_{D}\frac{P_{\text{CVCC},t}-P_{v,t}}{\rho u_{t}}, & \text{choked flow} \end{array}\right.$$

where $C_{D}$ is the volume discharge coefficient, which considers the nonuniform velocity profile at the nozzle, and ${\tilde{u}}_{t}$ is the corrected venting velocity. Based on ${\tilde{u}}_{t}$, the mass flow rate ${\dot{m}}_{v,t}$ of the venting can be calculated as ${\dot{m}}_{v,t}=A\rho {\tilde{u}}_{t}$, where A is the area of the safety valve orifice. For GGKNet, the rate of pressure change, $dP_{\text{in}}/dt$, during the pressure buildup process can be expressed as:

(11) $$\frac{dP_{\text{in}}}{dt}=\frac{RT}{V_{h}}\frac{dn}{dt}+\frac{nR}{V_{h}}\frac{dT}{dt}$$

where R is the universal gas constant, $T_{t}$ is the cell temperature, n represents the molar amount of gas accumulated in the headspace, and $V_{h}$ denotes the volume of the headspace. The change rate of the mole amount of gases dn/dt can be expressed as:

(12) $$\frac{dn}{dt}=\sum \frac{dn_{i}}{dt}=\sum \left(\frac{{\dot{m}}_{i}}{M_{i}}-\frac{{\dot{m}}_{v}Y_{i}}{M_{i}}\right)$$

where $M_{i}$ represents the molar mass of the gas component i. ${\dot{m}}_{i,t}$ is the mass change rate calculated via GGKNet, and $Y_{i,t}$ denotes the mass fraction of the gas component i in the headspace of the battery at that moment.

### 4.8 Statistical Analysis

The operando gas evolution tests based on the ARC‐GC platform were performed with three independent replicates (sample size n=3) to ensure reproducibility. In Figures 2B,C and 3 C, D, the data points for these gas evolution behaviors represent the calculated mean values of the three parallel experiments, with the error bars indicating the experimental error among the replicates. For the thermal data obtained from ARC, key thermal characteristic parameters including *T*_onset_, *T*_sc_ and *t*_sc_ are summarized in Table S6, which details both the raw replicate values and their corresponding calculated means and coefficients of variation (CV).

## Author Contributions

J. Z. and D. H. conceptualized the research. J. Z. and Z. H. supervised the project and acquired funding. Q. G. developed the operando gas diagnostic methods, curated the data, and conducted the investigation. S. Liu constructed the GGKNet, developed the gas generation mechanism, and validated the mechanism with support from J. Z. S. Li and X. K. conducted the gas‐sensing test by CVCC. J. Z., Q. G., S. Liu, and S. Li prepared the original draft and all authors contributed to the review and editing of the final manuscript.

## Conflicts of Interest

The authors declare no conflicts of interest.

## Supporting information


**Supporting  File**: advs76433‐sup‐0001‐SuppMat.pdf.

## Data Availability

The data and codes of this work has been open‐sourced in: https://doi.org/10.6084/m9.figshare.31208395.
