# Supplementary material for: Discovering Early‐Stage Gas Generation Kinetics Enables Thermal Runaway Early Warning in Lithium‐Ion Batteries
Source: Adv Sci (Weinh). 2026 Jul 9:e76433. Online ahead of print. doi: 10.1002/advs.76433 (PMC13348658; doi:10.1002/advs.76433)
Supplement: Supplementary file 1 — Supporting File: advs76433‐sup‐0001‐SuppMat.pdf. [file ADVS-9999-e76433-s001.pdf]

Supplementary Materials for
Discovering Early-Stage Gas Generation Kinetics Enables Thermal Runaway Early Warning in Lithium-ion Batteries

Jiabo Zhang[†]*, Qianzhen Guo[†], Shuaiqi Liu[†], Shuai Li,

Xianghao Kong, Dong Han*, Zhen Huang*

*Corresponding authors. Email: zhangjiabo@sjtu.edu.cn, dong_han@sjtu.edu.cn, z-huang@sjtu.edu.cn

[†]These authors contribute equally to this work.

This PDF file includes:

Materials and Methods

Figures S1 to S14

Tables S1 to S7

Materials and Methods

Operando gas diagnostics during TR

In this study, an ARC (BAC-90A, Hangzhou YOUNG Instruction Science & Technology Co., Ltd.) was employed to characterize the heat-generation behavior of lithium-ion batteries (LIBs), while the gases evolved during the heating process were sampled and quantified by gas chromatography (GC, Agilent 990 Micro GC, Agilent Technologies Inc.), and the schematic diagram of the testing platform is shown in Figure S1. Specifically, the platform primarily utilized the heat–wait–seek (H-W-S) protocol of the ARC to capture key thermal characteristic parameters such as the onset temperature of battery self-heating reactions (T_{onset}), the separator failure temperature (T_{sc}), and the time interval from the onset of heating to the occurrence of violent TR events (t_{sc}). To quantify the total gas yield, the safety valve of the test cell was pre-opened, and the battery was placed in a well-sealed stainless-steel vessel with an internal diameter of 80 mm and a height of 66 mm. The actual gas volume was then calculated based on the measured internal pressure of the vessel. Moreover, the vessel was equipped with dual gas ports: one connected to a pressure transducer (1 MPa full-scale range, 100 Hz sampling rate) for steady-state measurements, while the other interfaced with a high-frequency dynamic pressure transducer (20 MPa full-scale range, 100 Hz sampling rate) specifically designed to capture rapid pressure transients during explosion events. To enable gas sampling, the downstream end of one gas line was configured as a gas-sampling outlet and fitted in series with a filter and a pressure-reducing valve (PRV); the filter removes electrolyte solvent droplets, while the PRV ensures a constant 0.15 MPa outlet pressure during sampling.

On the other hand, to obtain temperature information of the cell, two type-N thermocouples were affixed to the center surface of the test cell. Due to the negligible temperature gradient inside small cylindrical cells before severe TR events [1, 2], the measured surface temperature can be regarded as the internal temperature of the cell. The first thermocouple was fed to the ARC with a sampling rate of 1 Hz to control its H-W-S program, whereas the second was connected to a data acquisition (DAQ) card with 100 Hz sampling rate for high-speed temperature monitoring. The detailed experimental operation steps are as follows:

I. Inside a nitrogen-filled glove box, the thermocouple-equipped cell and the stainless-steel vessel were placed together. The safety valve of the cell was then carefully opened, allowing

the cell to be transferred into the vessel. Once secured, the vessel was hermetically sealed and subsequently removed from the glove box.

II. After removal from the glove box, the vessel was placed inside the heating furnace of the ARC, and the two thermocouples were then connected - one to the ARC controller and the other to the DAQ card. Simultaneously, the gas ports were interfaced with corresponding pressure transducers to enable synchronous monitoring and recording of both temperature and pressure throughout the experiment.

III. After assembly, the system was allowed to stabilize for 30 minutes to facilitate the release of any residual gas trapped within the jelly-roll. Subsequently, the gas lines were back-purged with nitrogen to ensure an inert atmosphere.

IV. The H-W-S protocol of the ARC was employed to heat the sample cell, with an initial temperature step of 50°C and an increasing temperature step of 5°C. During the waiting phases, the system maintained a temperature rate sensitivity of 0.01 K min^{-1} to detect self-heating behavior.

V. At each target temperature during cell heating, a 5 mL gas sample was extracted from the system via gas-tight syringe. To ensure analytical reliability and eliminate systematic errors caused by residual gas in the sampling line from the previous temperature point, duplicate sampling was performed at each temperature point. The re-experimental validation of the gas sampling protocol is presented in Table S7. Each extracted sample was immediately injected into the micro-GC for compositional analysis, with the second measurement serving as the reference result.

It should be noted that preliminary experiments had confirmed the gas-sampling procedure during the HA stage did not affect the TR behavior of cells and that duplicate sampling represents the optimal sampling protocol, as shown in Figures S2 and S7. In addition to examining cell gas evolution behavior during the heat accumulation stage, supplementary tests were conducted to investigate gas generation before T_{onset}. Gas sampling was performed at the end of each temperature step in this test protocol. The temperature rise of the battery during the CA stage is controlled by the

H-W-S program of the ARC, so gas-sampling procedure do not alter the heating process. To prevent negative pressure buildup within the vessel resulting from gas extraction, high-purity nitrogen was systematically introduced to maintain atmospheric equilibrium. Furthermore, all formal tests under each SOC condition were performed in triplicate to ensure experimental reproducibility, and the reproducibility analysis of the thermal characteristic parameters is presented in Table S6. It can be observed that the coefficients of variation (CV) for most parameters remain below 10%, indicating satisfactory reproducibility.

Multidimensional sensing for early TR warning

Thermal abuse experiments were conducted on cells inside a multidimensional sensing platform to investigate gas generation, gas venting, jet flow and following combustion processes under confined conditions. The experimental platform integrated a CVCC, an optical Schlieren diagnostic system, and a time-resolved sensing system, enabling synchronized measurements of temperature, pressure, gas composition, and flow dynamics. Together, these diagnostics provide comprehensive, time-resolved characterization of the TR process from pre-vent pressure buildup to venting and post-vent combustion. A schematic of the test platform is provided in Figure S8. Detailed sensor specifications are provided in Tables S2, S3, and S4.

During each experiment, the test cell with 100% SOC was placed inside the CVCC and externally heated using a flexible resistive heating film mounted on the cell surface. The heating film was located entirely inside the CVCC, while its high-temperature insulated electrical leads were routed through an aviation connector to a direct current (DC) power supply positioned outside the chamber. Thermal abuse was applied under constant-power conditions. Moreover, two type-N thermocouples were installed inside the CVCC to monitor temperatures in real time, with one attached to the heating film and the other affixed to the cell surface. Gas composition was measured using two gas sensors targeting H_2 and CO, respectively. These sensors were mounted inside the CVCC to directly capture transient gas release and accumulation during TR. A pressure transducer was installed on a pressure tap connected to the chamber to monitor the internal gas pressure of the sealed enclosure. Sensor signals were then recorded and synchronized using a DAQ card controlled by a LabVIEW-based program. Note that, all thermocouples and gas sensors were connected via high-temperature insulated electrical leads and sealed aviation connectors to an external data acquisition system to

ensure gas-tight operation of the confined environment.

To visualize the gas venting and flow evolution during TR, a high-speed Schlieren diagnostic system was employed. The Schlieren system utilized two high-precision concave mirrors to generate a collimated light beam passing through the CVCC, forming a Z-shaped optical path. A knife-edge was positioned at the focal point to enhance sensitivity to refractive-index gradients, and a high-speed camera was aligned along the optical axis downstream of the knife-edge to capture Schlieren images. This configuration enabled visualization of high-speed gas jets, density gradients, and combustion-induced flow structures during valve opening and subsequent gas release. The detailed experimental procedure is summarized as follows:

I. Two gas sensors targeting H_2 and CO were mounted on a custom-designed fixture installed at the top of the CVCC. The sensitive surfaces of the sensors were oriented downward, directly facing the expected gas venting direction. The sensors were arranged with an relative angular separation of 120°, positioned at a vertical distance of 100 mm above the cell top and a radial distance of 80 mm from the cell centerline.

II. After mounting of gas sensors, a flexible resistive heating film was attached to the surface of the cylindrical cell using thermally conductive adhesive to ensure intimate contact, thereby providing circumferentially uniform thermal loading. Two thermocouples were attached to the cell surface to monitor the temperature evolution. One type-N thermocouple was placed between the heating film and the cell surface, while the other was placed on the opposite which is a narrow longitudinal gap without heating film covering. Both thermocouples were located at the axial mid-height of the cell and arranged with a relative angular separation of 180°. The heating film and thermocouples were fixed in place using metallic hose clamps to ensure stable positioning and good thermal contact during the tests.

III. Then, to secure the cell in the fixture, the instrumented cell was placed vertically into a V-shaped groove fixture located at the bottom of the CVCC. The fixture bolts were tightened to mechanically secure the cell and prevent displacement during gas venting and TR. The CVCC was subsequently sealed to establish a gas-tight confined environment.

IV. After the assembly of CVCC, data acquisition was initiated using a LabVIEW-based control program, and the gas sensors were powered on for a preheating period of approximately 2 h

to ensure signal stability. In parallel, the Schlieren optical system was aligned to ensure clear visualization of the test section.

V. Thermal abuse was applied by powering the heating film at a constant electrical power of 38 W until the end of TR. During the experiment, time-resolved signals of gas concentration, temperature, and chamber pressure were synchronously recorded. High-speed Schlieren imaging was performed simultaneously to capture the jet development and flow structures associated with gas venting and subsequent combustion.

VI. After the violent TR process, heating and optical recording was terminated. Sensor data acquisition was continued until the cell temperature returned to near ambient conditions, after which all experimental data were archived for post-processing and analysis.

Gas quantification method

In this study, the gas concentration evolution during cell heating was directly measured by GC. However, in the formulation of LIB TR mechanisms, it is necessary to quantify the amounts of individual gaseous species and distinguish between gases generated from side reactions and electrolyte vapors. Since the amount of nitrogen during the testing process is known, the actual gas amounts of other components can be calculated based on the nitrogen quantity combined with GC-measured concentration profiles.

At the nth sampling, the percentage of individual gaseous species i measured by GC is $\varphi_{i,n}$, i = N_2, CO_2, CO, H_2, CH_4, C_2H_4, C_2H_6. The molar quantity of each gas produced by side reactions, $M_{i,n}$, and the total gas production from side reactions, $M_{\mathrm{s},n}$, can be calculated using Eq. S1 and Eq. S2, respectively.

$$M_{i,n} = \frac{M_{\mathrm{N_2},n}}{\varphi_{\mathrm{N_2},n}}\varphi_{i,n}, (i = \mathrm{CO_2}, \mathrm{CO}, \mathrm{H_2}, \mathrm{CH_4}, \mathrm{C_2H_4}, \mathrm{C_2H_6}) \quad \text{(S1)}$$

$$M_{\mathrm{s},n} = \sum_i M_{i,n}, (i = \mathrm{CO_2}, \mathrm{CO}, \mathrm{H_2}, \mathrm{CH_4}, \mathrm{C_2H_4}, \mathrm{C_2H_6}) \quad \text{(S2)}$$

The total amount of gas within the vessel, M_n, including nitrogen, gas generated by side reactions, and electrolyte vapors, can be calculated using Eq. S3.

$$M_n = \frac{P_n V}{RT_n} = M_{s,n} + M_{N_2,n} + M_{e,n} \tag{S3}$$

where P_n and T_n are the pressure within the vessel and the cell temperature at the nth sampling. When $n = 0$, it represents the initial state of the test, with the temperature and pressure equilibrated to standard ambient conditions, and the vessel containing only nitrogen. Additionally, V is the actual volume of the gas, which can be calculated using Eqs. S4 and S5 [3], and R is the universal gas constant of 8.314 J mol^{-1} K^{-1}. $M_{N_2,n}$ and $M_{e,n}$ denote the amount of nitrogen and electrolyte vapors within the vessel at the nth sampling.

$$V = V_{vessel} - V_{LIB} + V_{void} \tag{S4}$$

$$V_{void} = 0.07V_{LIB} \tag{S5}$$

where V_{vessel} denotes the inner volume of the vessel, V_{LIB} denotes the volume of the cell, and V_{void} represents the void space within the test cell.

It should be noted that in calculating the actual production of individual gaseous species from side reactions at the nth sampling, the gas consumption during each sampling event, Δm_n, should be taken into account. This can be calculated using Eq. S6:

$$\Delta m_n = \begin{cases} \frac{P_n V_s}{RT_s}, & P_n \le P_s = 0.15 \text{ MPa} \\ \frac{P_s V_s}{RT_s}, & P_n > P_s = 0.15 \text{ MPa} \end{cases} \tag{S6}$$

where P_s and T_s denote the pressure and temperature at the gas sampling port, respectively. P_s was maintained at a constant value of 0.15 MPa, while T_s was held at ambient temperature (25°C). The sampled gas volume, V_s, was fixed at 5 mL.

When the nitrogen consumption due to sampling is considered, $M_{N_2,n}$ in Eq. S1 requires correction, resulting in the revised formulation of Eq.S7:

$$M_{N_2,n} = \frac{P_0 V}{RT_0} - \sum^{n-1} \Delta m_n \varphi_{N_2,n} \tag{S7}$$

For specific gas component i, the cumulative generated quantity at the nth sampling can be determined using Eq. S8.

$$M_{i,n,\text{total}} = M_{i,n} + \sum^{n-1} \Delta m_n \varphi_{i,n}, (i = \text{CO}_2, \text{CO}, \text{H}_2, \text{CH}_4, \text{C}_2\text{H}_4, \text{C}_2\text{H}_6) \quad \text{(S8)}$$

During the sampling process, the escape of electrolyte vapor is prevented by the filter. Consequently, the calculation of electrolyte vapor amount, $M_{\text{e},n}$, is unaffected by gas sampling and can be expressed by Eq. S9:

$$M_{\text{e},n} = \frac{P_n V}{R T_n} - \sum^{i} M_{i,n} - M_{\text{N}_2,n} \quad \text{(S9)}$$

In analyzing the corresponding explosion characteristics of battery venting gas (BVG), it is necessary to re-normalize the components of the gas generated within the cell. The specific component i can be calculated using the Eq. S10:

$$\varphi_{i,\text{venting}} = \frac{M_{i,\text{venting}}}{\sum M_{i,\text{venting}}}, (i = \text{CO}_2, \text{CO}, \text{H}_2, \text{CH}_4, \text{C}_2\text{H}_4, \text{C}_2\text{H}_6) \quad \text{(S10)}$$

where $M_{i,\text{venting}}$ represent the accumulated amounts of component i within the cell when the venting event occurs. They are obtained through linear interpolation of the experimentally measured values in relation to temperature.

The explosion characteristics of BVG at varied SOC conditions under normal temperature and pressure, including the lower explosion limit (LEL) and the upper explosion limit (UEL), are compared using Le Chatelier's (L-C) mixing rule, as shown in the following Eq. S11 [4]:

$$EL_{\text{BVG}} = \left[\sum \frac{\varphi_{i,\text{venting}}}{EL_i} \right]^{-1}, \quad \text{(S11)}$$

where EL_{BVG} is the explosion limit of BVG, EL_i is the explosion limit of the individual gas component i. Note that, BVG contains a large amount of inert gas CO_2. Therefore, CO_2 and H_2 in the BVG is treated as a new synthetic gas, replacing the original two components for calculation [4]. The EL_i of all gas components can be obtained from the literature [4, 5].

Three-dimensional computational fluid dynamic model of gas venting

In this research, to compare the predictive performance of GGKNet with that of other models, two other prediction models are employed: the fixed-composition-based model and the heat-generation-

based model. To ensure a fair comparison, all models are implemented on the same mesh and geometry. The boundary conditions for the three models are largely identical. For instance, the battery surface temperature uses experimental values as input, while constant temperature boundary conditions (25°C) are applied to the walls of CVCC. The differences in predictive performance among the three models solely reflect differences in their gas generation mechanisms.

1. Fixed-composition-based model: Consistent with previous studies [6], this model uses the gas composition ratio measured by GC after the thermal runaway of the battery as the boundary condition for the gas venting composition (Figures 6B and S4). Note that, after the thermal runaway, a large amount of electrolyte vapor has condensed into liquid, making it impossible to measure the amount of electrolyte vapor. Therefore, this model cannot account for the presence of electrolyte vapor. Additionally, this model cannot autonomously determine the gas venting velocity as an inlet boundary condition, often requiring an auxiliary venting model for modeling. To ensure consistency in boundary conditions as much as possible, the velocity calculated by GGKNet is used as the boundary condition here.

2. Heat-generation-based model: This model [7] first establishes a thermal model and calculates the reaction rates of various chemical reactions based on experimental temperature data:

$$\frac{dc_i}{dt} = -A_i c_i \exp\left(-\frac{Ea_i}{RT}\right) \tag{S12}$$

where c_i represents the normalized total amount of the substance i; A_i is the frequency factor; Ea_i is the activation energy; R is the molar gas constant, and T denotes the experimental temperature. Subsequently, these chemical reactions are linked to various gases, with the correlation coefficients calculated as Eq. S13:

$$\begin{pmatrix} c_{\mathrm{SEI},0} \\ c_{\mathrm{a},0} \\ c_{\mathrm{c},0} \\ c_{\mathrm{e},0} \\ c_{\mathrm{PVDF},0} \end{pmatrix}^T \begin{pmatrix} 0 & \omega_{\mathrm{SEI}} & 0 & 0 & \omega_{\mathrm{SEI}} & 0 \\ \omega_{\mathrm{a},1} & 0 & 0 & \omega_{\mathrm{a},2} & 0 & \omega_{\mathrm{a},3} \\ 0 & 0 & 0 & 0 & 0 & 0 \\ 0 & \omega_{\mathrm{e}} & 0 & 0 & 0 & 0 \\ 0 & 0 & \omega_{\mathrm{PVDF}} & 0 & 0 & 0 \end{pmatrix} = \begin{pmatrix} n_{\mathrm{CO}} \\ n_{\mathrm{CO_2}} \\ n_{\mathrm{H_2}} \\ n_{\mathrm{CH_4}} \\ n_{\mathrm{C_2H_4}} \\ n_{\mathrm{C_2H_6}} \end{pmatrix}^T \tag{S13}$$

where, the matrix $\omega_{i,j}$ is used to denote the amount of gas generated in different reactions, and n_i indicates the total gas production of gas i upon the conclusion of thermal runaway. Based on the

data obtained from GC, the matrix $\omega_{i,j}$ can be calculated and correlated with the chemical reaction rates in the thermal model to determine the gas generation rate and gas composition, which serve as the inlet boundary conditions.

Influence of additional chemical reactions on predictive performance of GGKNet

In this study, alongside the specification of chemical reactions in different regions based on existing expert knowledge, additional unspecified reactions were introduced to enable GGKNet to autonomously learn reactions without being constrained by prior knowledge. Taking the liquid electrolyte domain as an example, the number of these additional reactions was varied among 3, 4, and 5, with training results presented in Figure S14. It is observed that a relatively low number of additional reactions (n=3) leads to high system stiffness, making training prone to significant oscillations. Conversely, a relatively large number of additional reactions (n=5) may result in insufficient training under unchanged hyperparameters, necessitating an increased learning rate to achieve better convergence. Furthermore, an excessively low stiffness often diminishes the generalization ability of GGKNet, leading to overfitting. Therefore, for the number of additional reactions, a relatively balanced value should be selected.

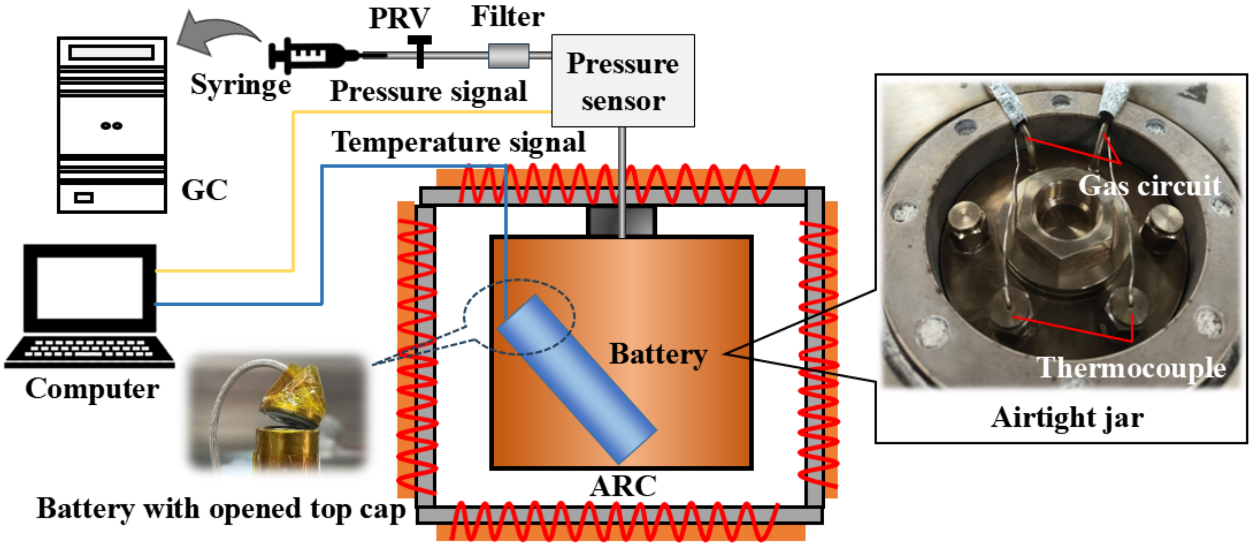

Figure S1: Schematic diagram of the operando gas diagnostics platform.

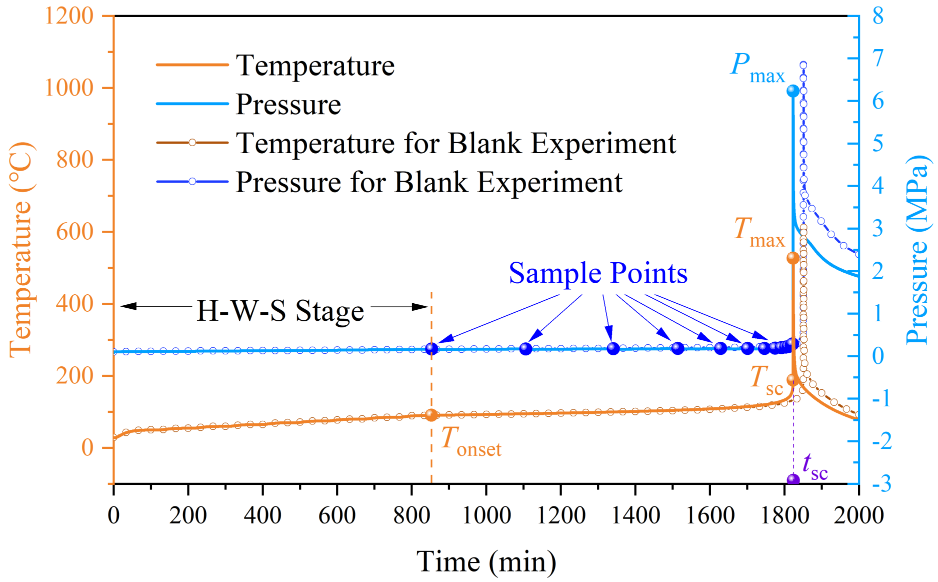

Figure S2: Evolution of cell surface temperature and internal vessel pressure: gas-sampling vs. non-sampling conditions.

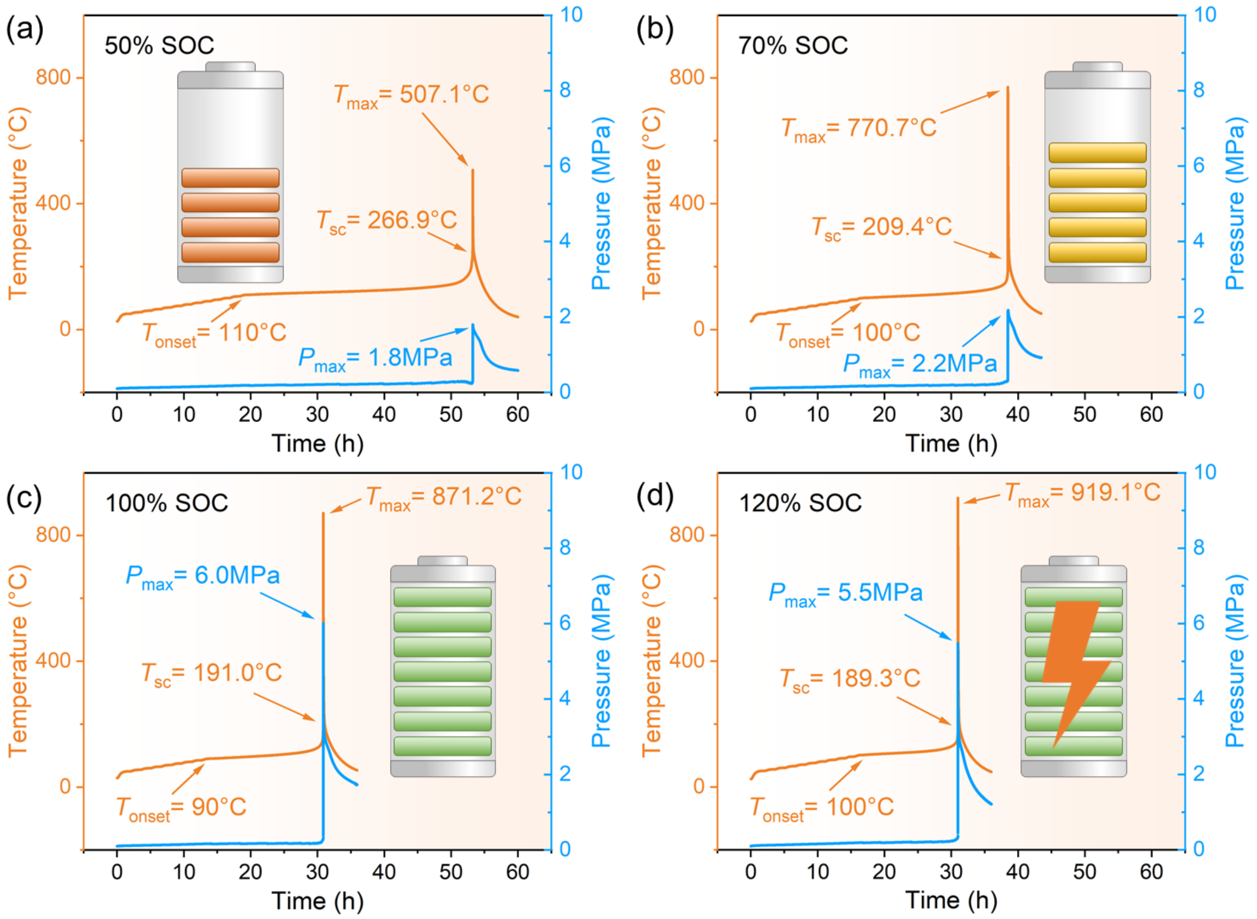

Figure S3: Evolution of cell temperature and internal vessel pressure for cells with different SOCs, with T_{onset}, T_{sc}, T_{max}, and P_{max} labeled: (a) 50% SOC , (b) 70% SOC , (c) 100% SOC, (d) 120% SOC.

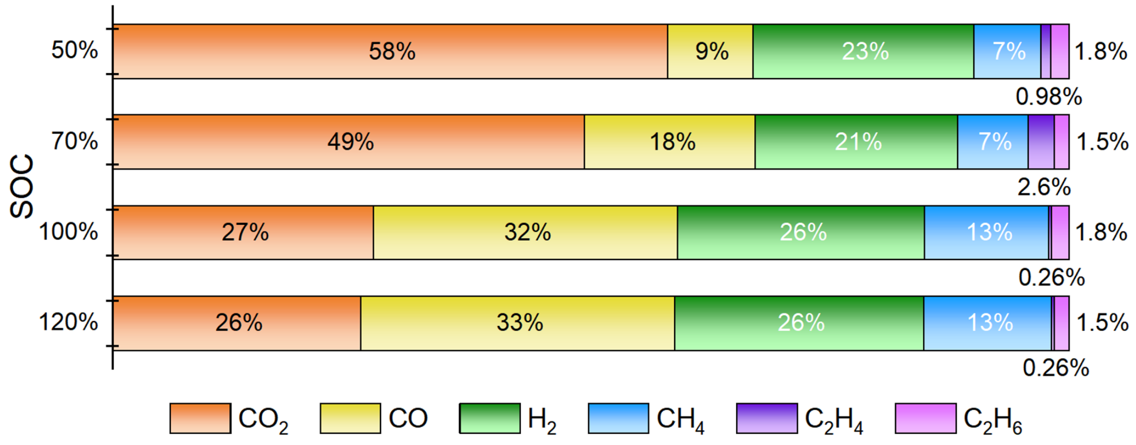

Figure S4: Post-TR gas composition for LIBs at 50%/70%/100%/120% SOC.

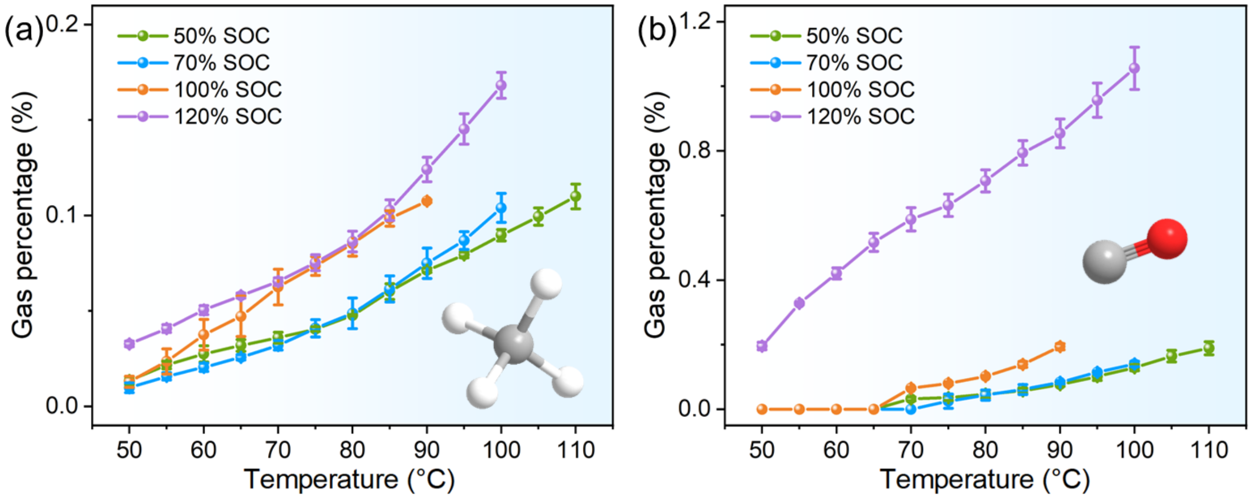

Figure S5: The gas components evolution during the chemical activation stage for LIBs at 50%/70%/100%/120% SOC: (a) CH_4; (b) CO.

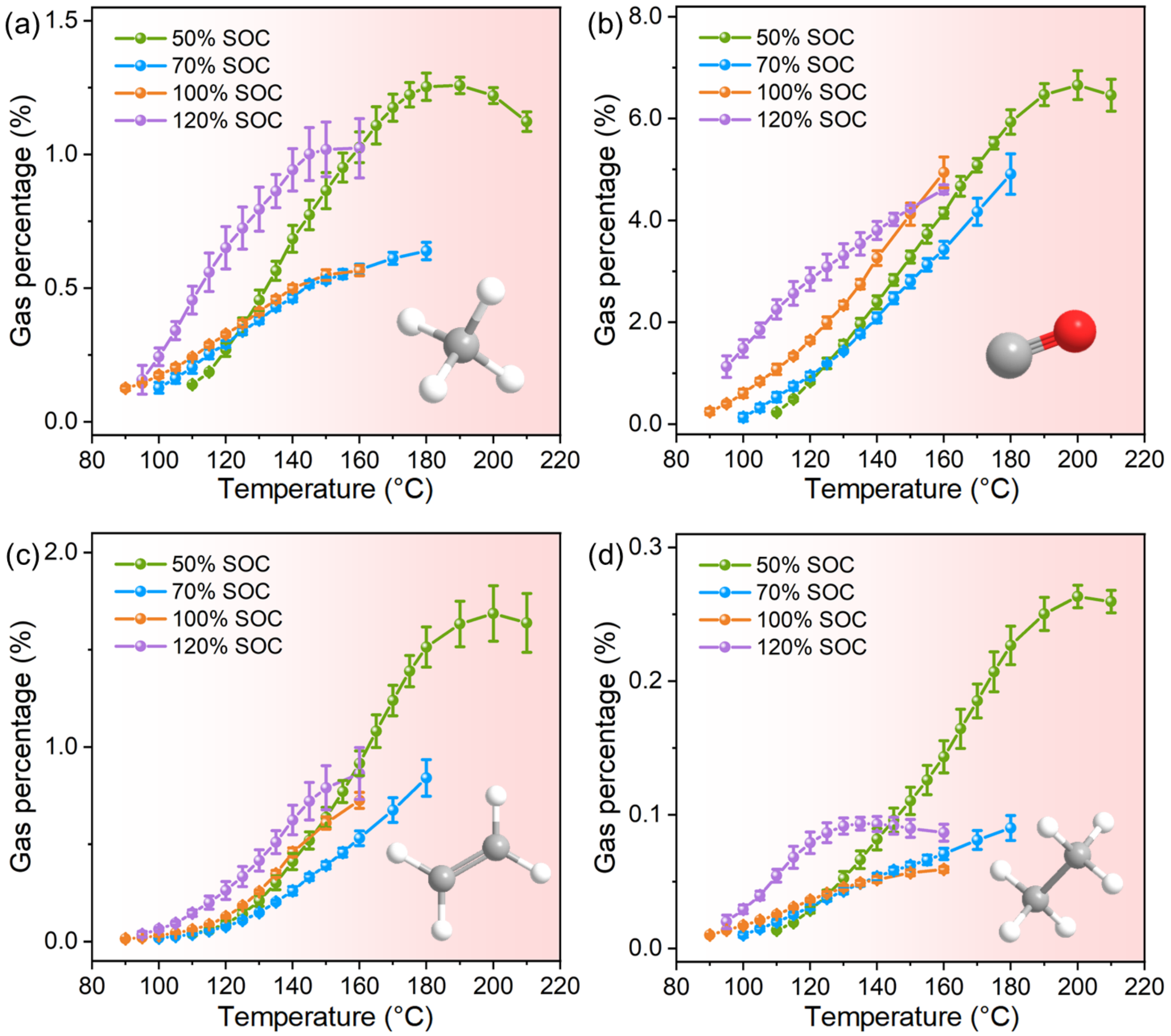

Figure S6: The gas component evolution during the heat accumulation stage for LIBs at 50%/70%/100%/120% SOC: (a) CH_4; (b) CO; (c) C_2H_4; (d) C_2H_6.

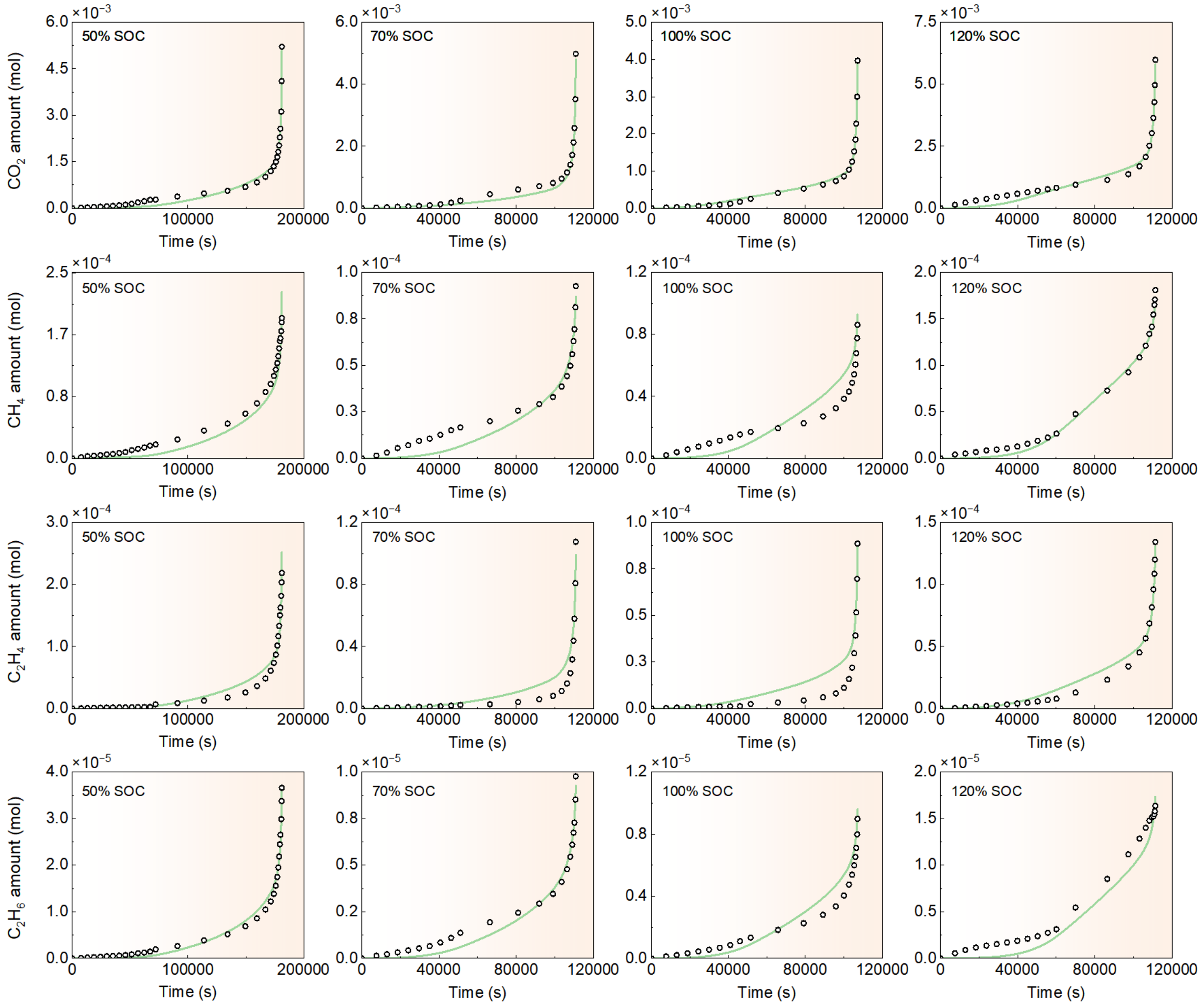

Figure S7: The comparison of predicted gas component (CO_2, CH_4, C_2H_4, C_2H_6) evolution and experiment results at 50%/70%/100%/120% SOC.

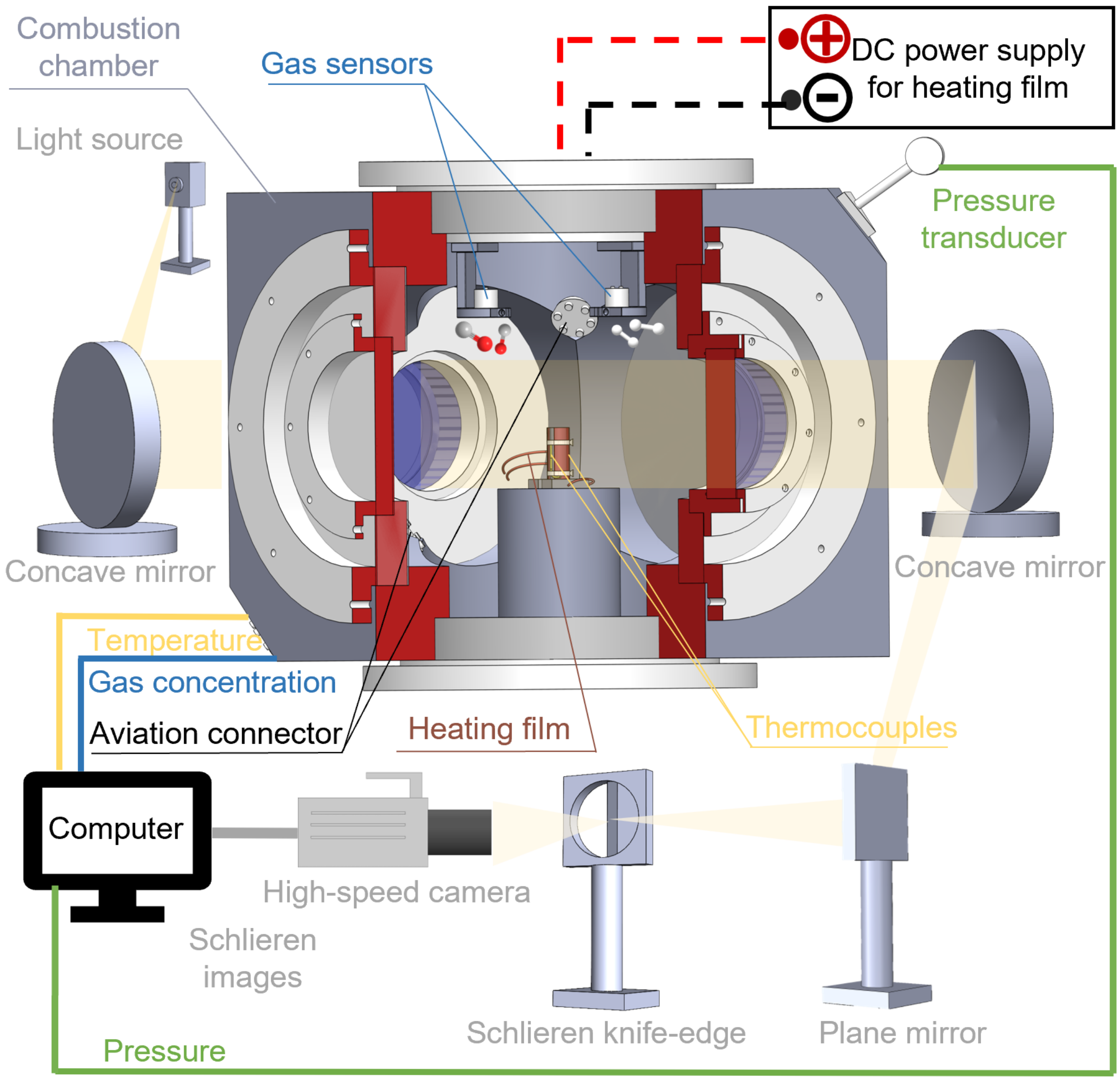

Figure S8: Schematic diagram of the multidimensional sensing platform composed of a constant volume combustion chamber, an optical Schlieren diagnostic system, and a time-resolved sensing system.

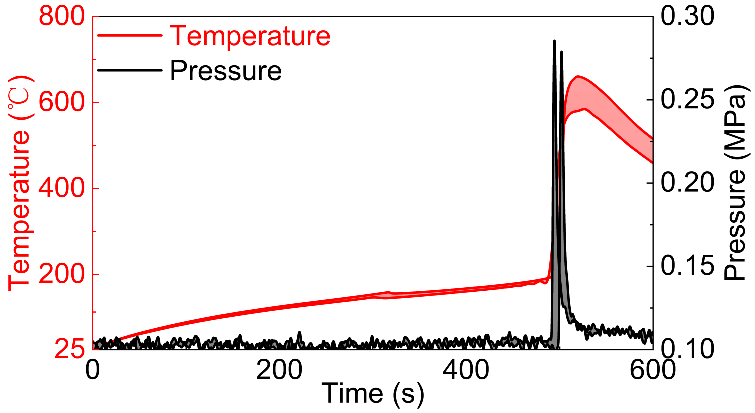

Figure S9: Evolution of cell surface temperature and pressure within the optical constant volume combustion chamber in two independent experiments.

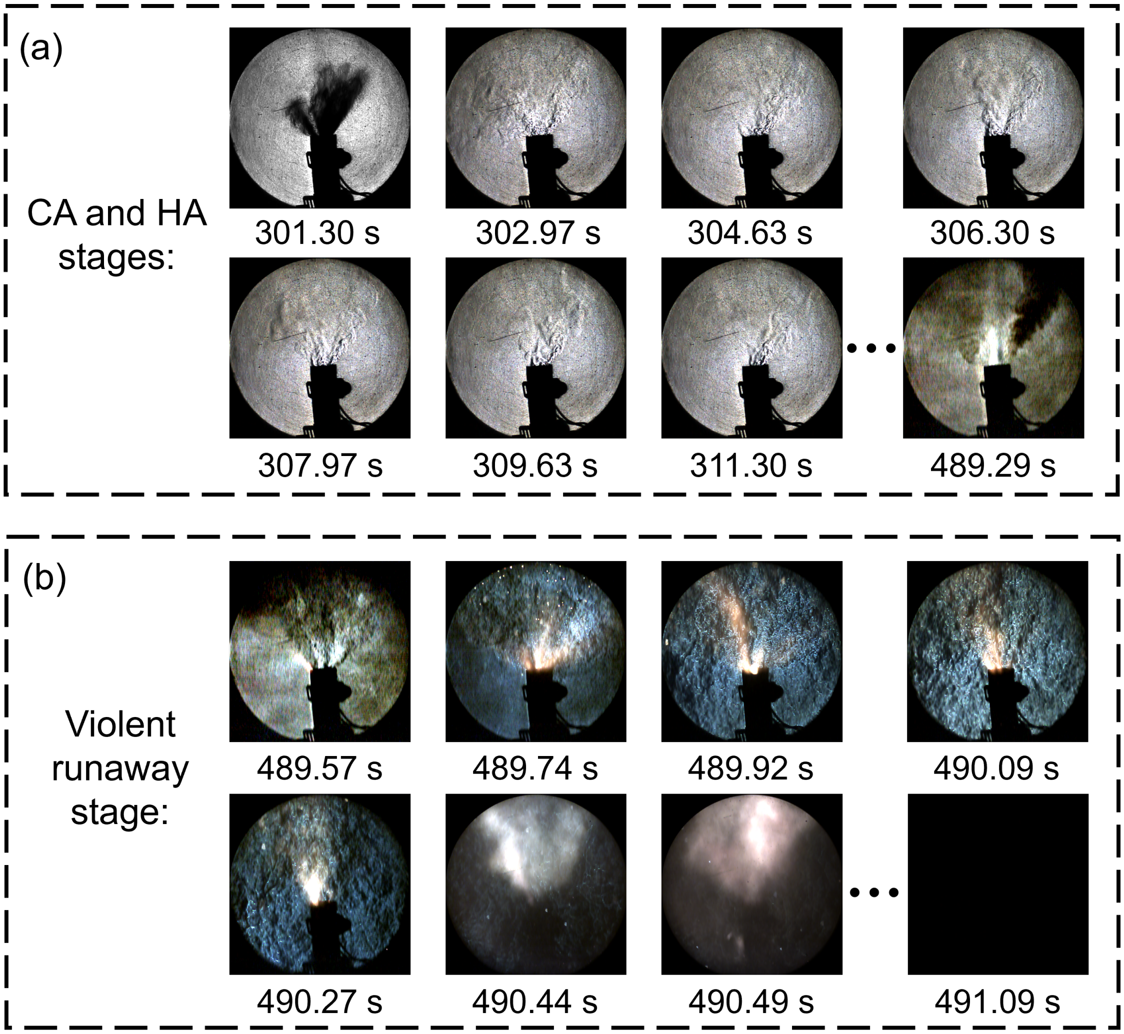

Figure S10: The Schlieren images from gas venting to combustion for cell inside the optical constant volume combustion chamber.

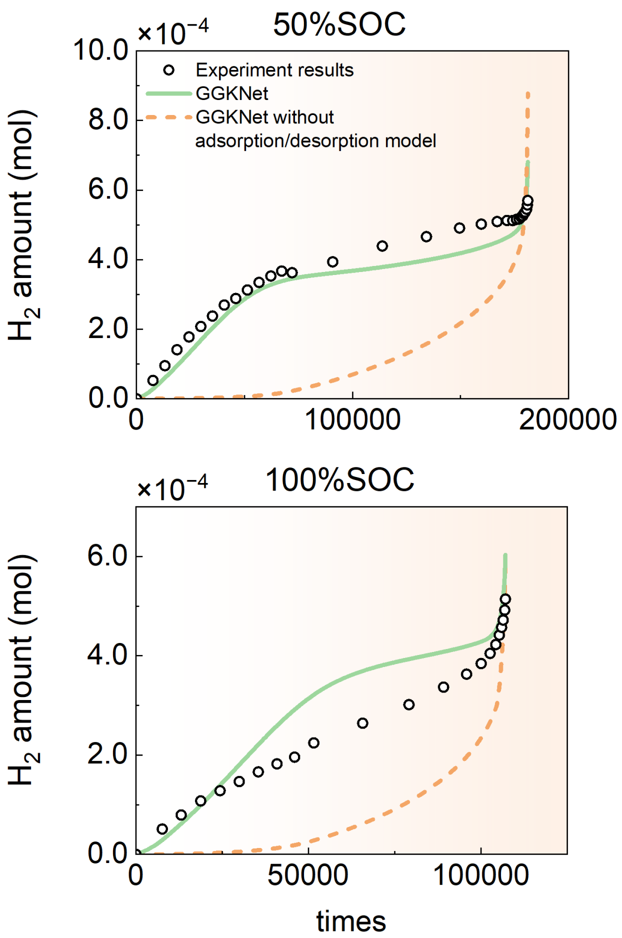

Figure S11: Comparison of the impact of hydrogen adsorption-desorption models on the predictive performance of GGKNet under different SOCs.

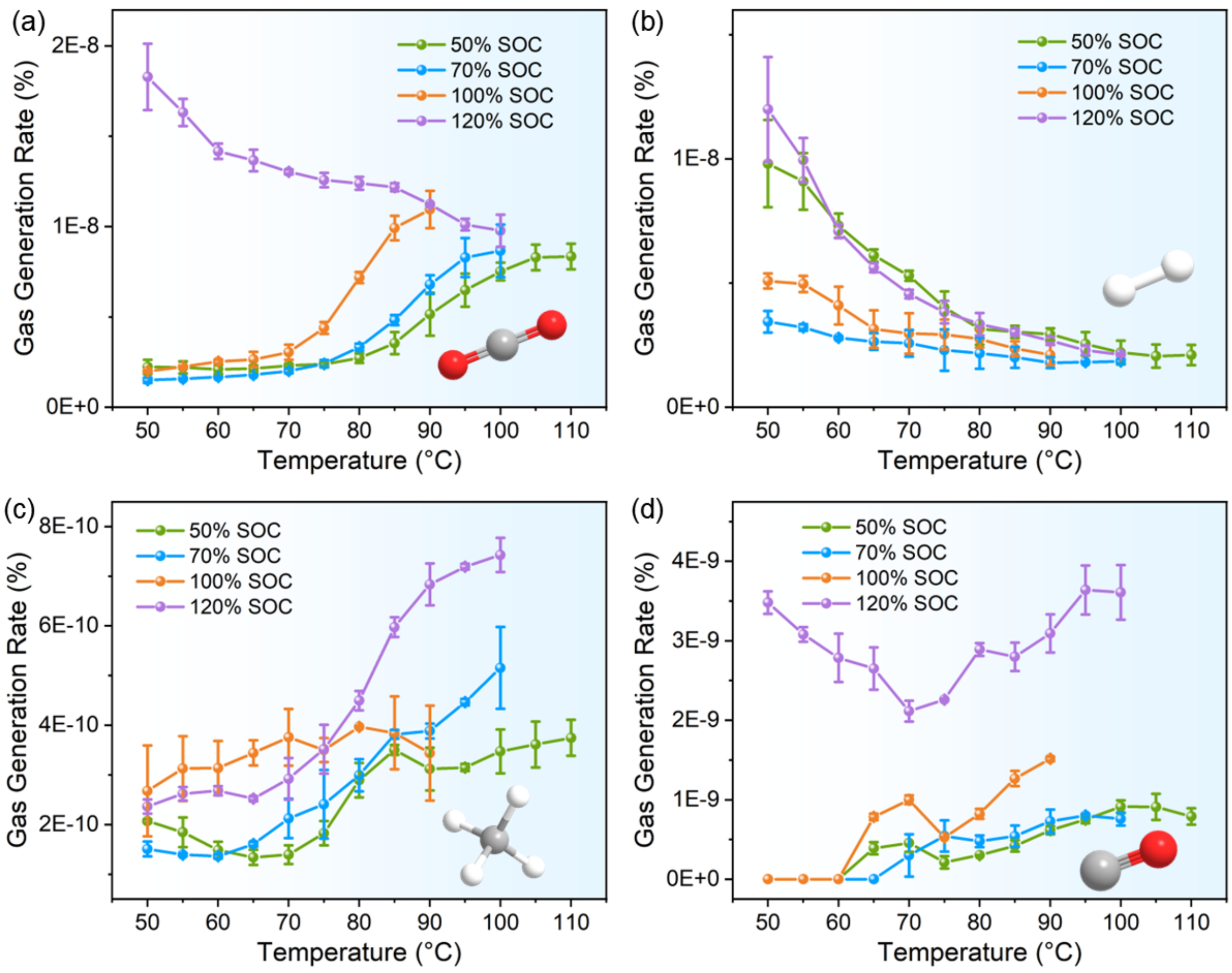

Figure S12: Temperature-dependent gas generation rates during the chemical activation stage for LIBs at 50%/70%/100%/120% SOC: (a) CO_2; (b) H_2; (c) CH_4; (d) CO.

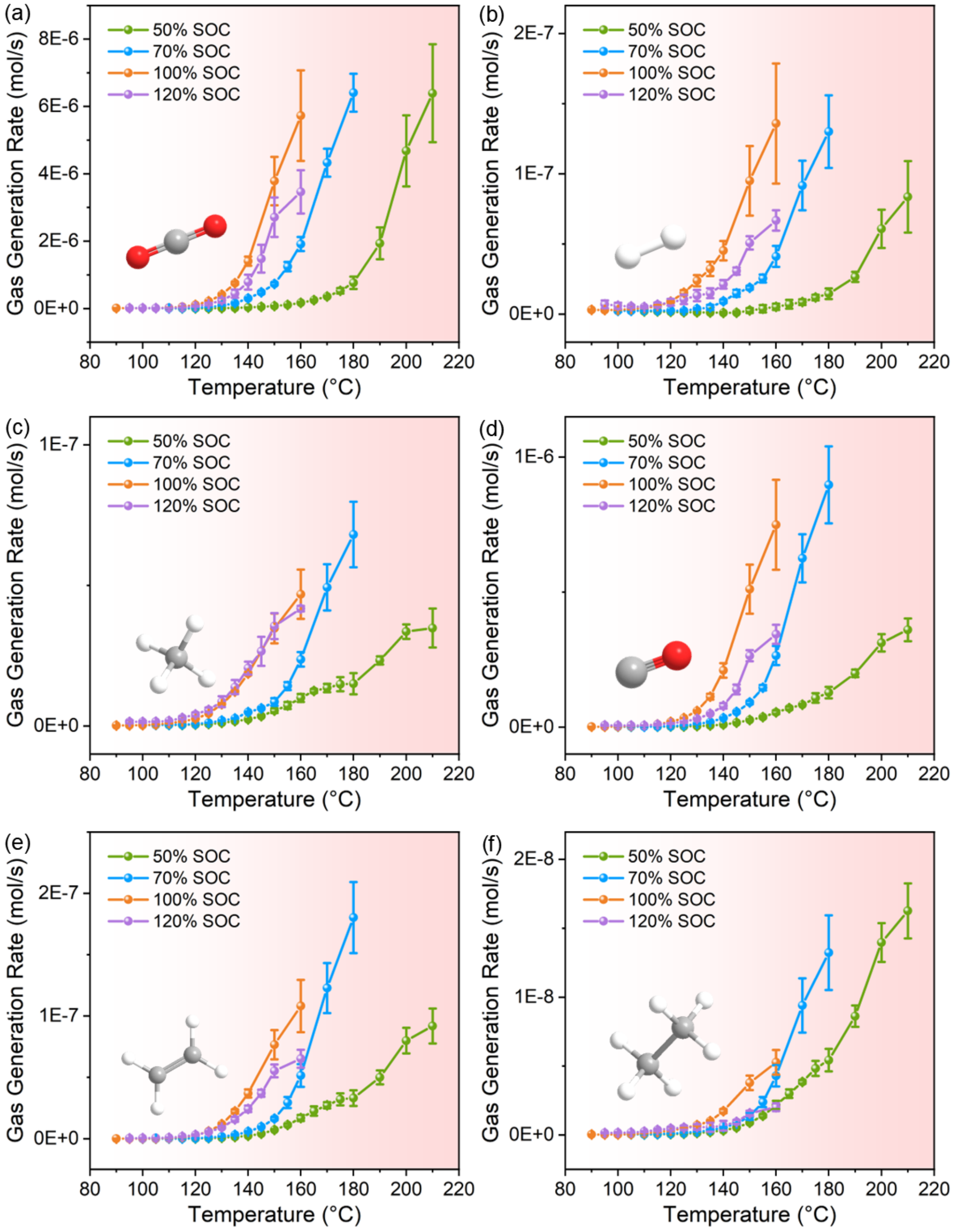

Figure S13: Temperature-dependent gas generation rates during the heat accumulation stage for LIBs at 50%/70%/100%/120% SOC: (a) CO_2; (b) H_2; (c) CH_4; (d) CO; (e) C_2H_4; (f) C_2H_6.

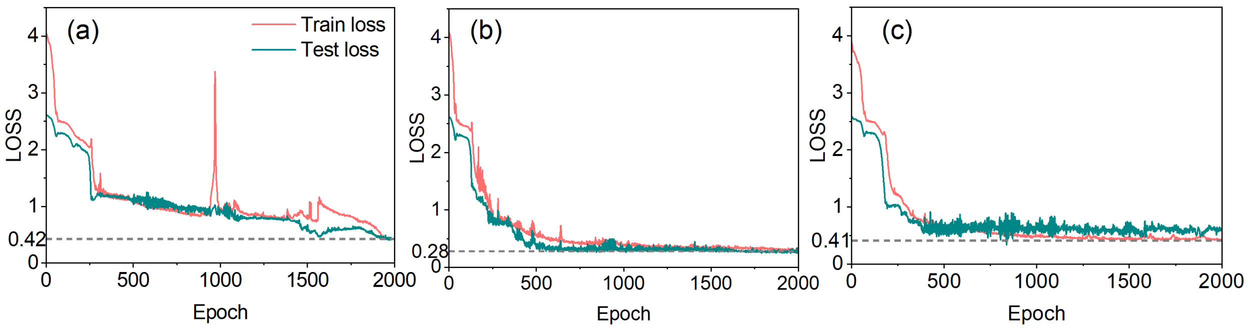

Figure S14: Training results of GGKNet with different additional reactions number, *n*, of the liquid electrolyte domain: (a) *n*=3; (b) *n*=4; (c) *n*=5.

Table S1: Summary of the test cell parameters.

Item	Specification
Dimensions (mm)	18 (diameter) × 65 (height)
Naked cell weight (g)	41.63 ± 0.10
Specific heat capacity ($J \cdot kg^{-1} \cdot K^{-1}$)	944.80
Cathode material	$LiNi_{0.8}Co_{0.1}Mn_{0.1}O_2$
Anode material	Graphite
Electrolyte solvents	EMC: DMC: EC
Salt	$LiPF_6$
Rated capacity (Ah)	2.85 ± 0.05
Nominal voltage (V)	3.65 ± 0.05
Energy density ($Wh \cdot kg^{-1}$)	249.88 ± 0.03
Number of safety valve holes	5

Table S2: The main parameters of constant volume combustion chamber for thermal abuse experiments.

Category	Parameter	Value / Description
Combustion chamber	Material	Steel
	Main shape	Intersection of three orthogonal cylinders (300 mm diameter) in a 500 mm edge-length cube
	Volume	50 L
	Initial gas	Air (room temperature, 1 atm)
Optical windows	Material	Quartz glass
	Position	Diametrically opposed to form a linear optical path
	Diameter	132 mm
Heating method	Material	Polyimide-encapsulated flexible resistive heating film
	Dimensions	53 mm × 64 mm
	Power	38 W

Table S3: Components and specifications of the optical Schlieren diagnostic system.

Component	Quantity	Specifications
Lens of camera	1	LAOWA 100 mm, F2.8
Light source	1	Luftvis-LED-35 W
Plane mirror	1	Luftvis-160, 75 mm diameter
Concave mirror	2	Luftvis-160, 1300 mm focal length, 160 mm diameter
Schlieren knife-edge	1	Luftvis-160
High-speed camera	1	Model: Revealer S1310C<br>Frame rate: 1000 FPS<br>Temporal resolution: 1 ms<br>Exposure time: 20 μs<br>Image resolution: 704 × 680 pixels<br>Spatial resolution: 0.185 mm/pixel

Table S4: The main parameters of the time-resolved sensing system for thermal abuse experiments inside the CVCC.

Signal	Testing method	Sampling rate	Full-scale range	Error range
Pressure	Pressure transducer (HELM HM90)	100 Hz	0~5 MPa	±0.1%
Temperature	Type-N thermocouples (Omega 0.8 mm diameter)	10 Hz	-50~700°C	±0.75%
H_2	Electrochemical gas sensor (Sangbay S4H_2-1000F)	1 Hz	1~1000 ppm	±2%
CO	Electrochemical gas sensor (Semeatech 7-CO-10000)	1 Hz	1~10000 ppm	±3%

Table S5: Chemical reactions and kinetic parameters learned by GGKNet

No.	Domain	E_a(kJ/mol)	b	$\ln A$	Chemical Reaction
LR1	Cathode	77.69	0.27	0.99	$0.07\,Li + 0.15\,LIB \longrightarrow 0.02\,S_1 + 0.02\,S_2 + 0.02\,S_3 + 0.04\,S_4 + 0.02\,S_9 + 0.02\,S_1 + 0.02\,S_{11}$
LR2	Cathode	90.57	0.62	3.45	$0.12\,Li + 0.46\,LIB \longrightarrow 0.09\,CO + 0.05\,S_1 + 0.04\,S_2 + 0.04\,S_3 + 0.04\,S_4 + 0.06\,S_9 + 0.04\,S_{10} + 0.05\,S_{11} + 0.05\,S_{12}$
LR3	Cathode	73.64	0.54	2.77	$0.17\,LIB \longrightarrow 0.02\,S_1 + 0.02\,S_2 + 0.02\,S_3 + 0.02\,S_4 + 0.03\,S_9 + 0.03\,S_{10} + 0.02\,S_{11}$
LR4	Cathode	58.41	0.00	0.49	$0.06\,Li + 0.01\,LIB + 0.62\,S_{12} \longrightarrow 0.04\,CO_2 + 0.03\,C_2H_4 + 0.02\,C_2H_6 + 0.04\,CH_4 + 0.02\,CO + 0.07\,S_1 + 0.07\,S_2 + 0.07\,S_3 + 0.07\,S_4 + 0.07\,S_9 + 0.07\,S_{10} + 0.07\,S_{11}$
LR5	Cathode	68.03	0.01	0.04	$0.05\,Li + 0.07\,S_1 + 0.07\,S_2 + 0.07\,S_3 + 0.07\,S_4 + 0.07\,S_{10} + 0.07\,S_{11} \longrightarrow 0.02\,S_9 + 0.42\,S_{12}$
LR6	Cathode	56.35	0.00	0.12	$0.06\,Li + 0.62\,S_{12} \longrightarrow 0.04\,CO_2 + 0.02\,C_2H_4 + 0.02\,C_2H_6 + 0.06\,CH_4 + 0.02\,CO + 0.06\,S_1 + 0.06\,S_2 + 0.06\,S_3 + 0.06\,S_4 + 0.06\,S_9 + 0.06\,S_{10} + 0.06\,S_{11}$
LR7	Cathode	59.32	0.00	0.41	$0.05\,Li + 0.61\,S_{12} \longrightarrow 0.04\,CO_2 + 0.03\,C_2H_4 + 0.02\,C_2H_6 + 0.03\,CH_4 + 0.04\,CO + 0.07\,S_1 + 0.07\,S_2 + 0.06\,S_3 + 0.06\,S_4 + 0.06\,S_9 + 0.06\,S_{10} + 0.06\,S_{11}$
LR8	Anode	74.16	1.11	3.63	$0.51\,Li + 0.23\,LIB \longrightarrow 0.2\,CO_2 + 0.03\,S_{12}$
LR9	Anode	83.98	0.67	3.93	$0.35\,Li + 0.3\,LIB \longrightarrow 0.16\,C_2H_4 + 0.04\,S_6 + 0.05\,S_7 + 0.05\,S_8$

Table S5: Chemical reactions and kinetic parameters learned by GGKNet (Continued)

No.	Domain	E_a(kJ/mol)	b	ln A	Chemical Reaction
LR10	Anode	84.14	0.35	1.58	$0.11\,Li + 0.27\,LIB \longrightarrow 0.01\,CO_2 + 0.04\,S_5 + 0.03\,S_6 + 0.04\,S_7 + 0.06\,S_8 + 0.03\,S_9 + 0.03\,S_{10} + 0.03\,S_{11} + 0.01\,S_{12}$
LR11	Anode	85.05	0.35	1.87	$0.28\,Li + 0.42\,LIB \longrightarrow 0.04\,S_5 + 0.06\,S_6 + 0.04\,S_7 + 0.05\,S_8 + 0.07\,S_9 + 0.05\,S_{10} + 0.04\,S_{11} + 0.08\,S_{12}$
LR12	Anode	90.59	0.48	2.76	$0.11\,Li + 0.48\,LIB \longrightarrow 0.07\,CO_2 + 0.06\,S_5 + 0.06\,S_6 + 0.04\,S_7 + 0.05\,S_8 + 0.05\,S_9 + 0.08\,S_{10} + 0.05\,S_{11} + 0.04\,S_{12}$
LR13	Anode	80.86	0.22	2.21	$0.23\,Li + 0.08\,LIB + 0.03\,S_7 + 0.02\,S_8 + 0.2\,S_{12} \longrightarrow 0.02\,CO_2 + 0.03\,C_2H_4 + 0.05\,C_2H_6 + 0.03\,CH_4 + 0.04\,CO + 0.03\,S_5 + 0.03\,S_6 + 0.04\,S_9 + 0.03\,S_{10} + 0.03\,S_{11}$
LR14	Anode	66.66	0.31	3.17	$0.43\,Li + 0.18\,LIB \longrightarrow 0.01\,H_2 + 0.16\,CO + 0.01\,S_{12}$
LR15	Anode	55.98	0.01	0.62	$0.08\,Li + 0.51\,S_{12} \longrightarrow 0.03\,CO_2 + 0.09\,CH_4 + 0.01\,CO + 0.06\,S_5 + 0.06\,S_6 + 0.02\,S_7 + 0.06\,S_8 + 0.06\,S_9 + 0.06\,S_{10} + 0.06\,S_{11}$
LR16	Anode	61.90	0.00	0.23	$0.09\,Li + 0.07\,S_5 + 0.08\,S_6 + 0.08\,S_7 + 0.09\,S_8 + 0.04\,S_{10} + 0.05\,S_{11} \longrightarrow 0.01\,S_9 + 0.41\,S_{12}$
LR17	Electrolyte(l)	92.04	0.25	2.56	$0.07\,Li + 0.3\,LIB \longrightarrow 0.05\,C_2H_4 + 0.04\,S_9 + 0.06\,S_{10} + 0.04\,S_{11} + 0.1\,S_{12}$
LR18	Electrolyte(l)	87.57	0.18	2.76	$0.09\,Li + 0.41\,LIB \longrightarrow 0.11\,S_9 + 0.1\,S_{10} + 0.07\,S_{11} + 0.12\,S_{12}$
LR19	Electrolyte(l)	96.03	0.27	3.65	$0.07\,Li + 0.36\,LIB \longrightarrow 0.09\,C_2H_6 + 0.06\,S_9 + 0.06\,S_{10} + 0.12\,S_{11} + 0.04\,S_{12}$

Table S5: Chemical reactions and kinetic parameters learned by GGKNet (Continued)

No.	Domain	E_a(kJ/mol)	b	ln A	Chemical Reaction
LR20	Electrolyte(l)	84.28	0.21	1.19	$0.03\,Li + 0.14\,LIB \longrightarrow 0.04\,S_9 + 0.04\,S_{10} + 0.05\,S_{11} + 0.01\,S_{12}$
LR21	Electrolyte(l)	62.95	0.02	0.13	$0.05\,Li + 0.25\,S_{12} \longrightarrow 0.02\,CO_2 + 0.03\,CO + 0.06\,S_9 + 0.06\,S_{10} + 0.06\,S_{11}$
LR22	Electrolyte(l)	62.61	0.00	0.22	$0.07\,Li + 0.34\,S_{12} \longrightarrow 0.05\,CO_2 + 0.01\,C_2H_6 + 0.03\,CO + 0.08\,S_9 + 0.08\,S_{10} + 0.08\,S_{11}$
LR23	Electrolyte(l)	62.86	0.00	0.23	$0.06\,Li + 0.49\,S_{12} \longrightarrow 0.07\,CO_2 + 0.04\,C_2H_4 + 0.03\,C_2H_6 + 0.04\,CH_4 + 0.05\,CO + 0.08\,S_9 + 0.08\,S_{10} + 0.08\,S_{11}$
LR24	Electrolyte(l)	62.81	0.00	0.00	$0.07\,Li + 0.44\,S_{12} \longrightarrow 0.07\,CO_2 + 0.02\,C_2H_4 + 0.02\,C_2H_6 + 0.03\,CH_4 + 0.05\,CO + 0.08\,S_9 + 0.08\,S_{10} + 0.08\,S_{11}$
LR25	Electrolyte(l)	61.13	0.00	0.08	$0.08\,Li + 0.5\,S_{12} \longrightarrow 0.07\,CO_2 + 0.03\,C_2H_4 + 0.03\,C_2H_6 + 0.06\,CH_4 + 0.05\,CO + 0.09\,S_9 + 0.09\,S_{10} + 0.09\,S_{11}$
LR26	Electrolyte(l)	51.91	0.02	0.25	$0.13\,Li + 0.41\,S_{12} \longrightarrow 0.03\,CO_2 + 0.01\,C_2H_6 + 0.11\,CH_4 + 0.02\,CO + 0.08\,S_9 + 0.08\,S_{10} + 0.08\,S_{11}$
LR27	Electrolyte(l)	63.40	0.00	0.01	$0.06\,Li + 0.44\,S_{12} \longrightarrow 0.07\,CO_2 + 0.03\,C_2H_4 + 0.02\,C_2H_6 + 0.02\,CH_4 + 0.06\,CO + 0.08\,S_9 + 0.08\,S_{10} + 0.08\,S_{11}$
LR28	Electrolyte(l)	62.45	0.00	0.05	$0.07\,Li + 0.49\,S_{12} \longrightarrow 0.08\,CO_2 + 0.06\,C_2H_4 + 0.05\,C_2H_6 + 0.06\,CH_4 + 0.07\,CO + 0.09\,S_9 + 0.09\,S_{10}$
LR29	Electrolyte(g)	89.16	0.41	1.94	$0.27\,ELE(g) \longrightarrow 0.02\,C_2H_4 + 0.1\,S_9 + 0.05\,S_{10} + 0.04\,S_{11} + 0.06\,S_{12}$

Table S5: Chemical reactions and kinetic parameters learned by GGKNet (Continued)

No.	Domain	E_a(kJ/mol)	b	$\ln A$	Chemical Reaction
LR30	Electrolyte(g)	93.49	0.56	3.14	$0.33\,ELE(g) \longrightarrow 0.13\,S_9 + 0.04\,S_{10} + 0.05\,S_{11} + 0.11\,S_{12}$
LR31	Electrolyte(g)	95.19	0.71	4.07	$0.27\,ELE(g) \longrightarrow 0.06\,S_9 + 0.06\,S_1 + 0.10\,S_{11} + 0.04\,S_{12}$
LR32	Electrolyte(g)	52.18	0.00	0.16	$0.03\,ELE(g) \longrightarrow 0.02\,S_9$
LR33	Electrolyte(g)	63.43	0.00	0.23	$0.52\,S_{12} \longrightarrow 0.08\,CO_2 + 0.05\,C_2H_4 + 0.03\,C_2H_6 + 0.03\,CH_4 + 0.06\,CO + 0.09\,S_9 + 0.09\,S_1 + 0.09\,S_{11}$
LR34	Electrolyte(g)	65.25	0.04	0.58	$0.24\,S_{12} \longrightarrow 0.03\,CO_2 + 0.02\,CO + 0.06\,S_9 + 0.06\,S_{10} + 0.06\,S_{11}$
LR35	Electrolyte(g)	60.36	0.00	0.12	$0.59\,S_{12} \longrightarrow 0.08\,CO_2 + 0.05\,C_2H_4 + 0.03\,C_2H_6 + 0.07\,CH_4 + 0.07\,CO + 0.1\,S_9 + 0.1\,S_{10} + 0.1\,S_{11}$
LR36	Electrolyte(g)	67.53	0.00	0.11	$0.31\,S_{12} \longrightarrow 0.05\,CO_2 + 0.02\,C_2H_4 + 0.02\,C_2H_6 + 0.03\,CO + 0.06\,S_9 + 0.06\,S_{10} + 0.06\,S_{11}$

Table S6: Reproducibility analysis of TR thermal characteristic parameters for LIBs.

SOC	Parameter	Exp 1	Exp 2	Exp 3	Mean	CV
50%	T_{onset} (°C)	110.5	110.4	110.4	110.4	0.05%
	T_{sc} (°C)	268.0	257.3	266.9	264.1	2.23%
	t_{sc} (min)	2819	3028	3193	3013	6.22%
70%	T_{onset} (°C)	105.6	100.4	100.5	102.2	2.91%
	T_{sc} (°C)	207.2	213.6	209.4	210.1	1.55%
	t_{sc} (min)	2009	2201	2308	2173	6.97%
100%	T_{onset} (°C)	90.53	90.46	90.46	90.48	0.04%
	T_{sc} (°C)	193.1	188.4	191.0	190.8	1.23%
	t_{sc} (min)	1787	1823	1851	1820	1.76%
120%	T_{onset} (°C)	101.7	101.7	96.53	99.98	2.99%
	T_{sc} (°C)	189.3	188.6	193.6	190.5	1.42%
	t_{sc} (min)	1859	1538	1602	1666	10.2%

Table S7: Pre-experimental validation of the gas sampling protocol.

Temperature	Gas species	Sampling 1	Sampling 2	Sampling 3
80°C	CO_2	1.232%	1.598%	1.603%
	H_2	1.445%	1.911%	1.952%
	CH_4	1.283%	1.591%	1.619%
	CO	2.307%	2.835%	2.851%
85°C	CO_2	1.848%	2.544%	2.596%
	H_2	1.703%	2.154%	2.149%
	CH_4	1.613%	1.771%	1.770%
	CO	2.993%	3.545%	3.560%

References

[1] P. Zhao, L. Liu, L. Zhang, Y. Chen, Mitigating battery thermal runaway through mild combustion. *Chemical Engineering Journal Advances* **9**, 100208 (2022).

[2] Q. Guo, J. Zhang, C. Zhou, Z. Huang, D. Han, Thermal runaway behaviors and kinetics of NCM Lithium-ion batteries at different heat dissipation conditions. *Journal of The Electrochemical Society* **170** (8), 080507 (2023).

[3] P. Qin, J. Sun, Q. Wang, A new method to explore thermal and venting behavior of lithium-ion battery thermal runaway. *Journal of power sources* **486**, 229357 (2021).

[4] L. Xu, *et al.*, Thermal runaway propagation behavior and gas production characteristics of NCM622 battery modules at different state of charge. *Process Safety and Environmental Protection* **185**, 267–276 (2024).

[5] A. Z. Mendiburu, J. A. Carvalho Jr, Y. Ju, Flammability limits: a comprehensive review of theory, experiments, and estimation methods. *Energy & Fuels* **37** (6), 4151–4197 (2023).

[6] B. Mao, C. Zhao, H. Chen, Q. Wang, J. Sun, Experimental and Modeling Analysis of Jet Flow and Fire Dynamics of 18650-Type Lithium-Ion Battery. *Applied Energy* **281**, 116054 (2021).

[7] G. Wang, *et al.*, Revealing Particle Venting of Lithium-Ion Batteries during Thermal Runaway: A Multi-Scale Model toward Multiphase Process. *eTransportation* **16**, 100237 (2023).
